# Integrated gene expression profiles reveal a transcriptomic network underlying the thermogenic response in adipose tissue

**DOI:** 10.1038/s41598-023-33367-w

**Published:** 2023-05-04

**Authors:** Jordi Rodó, Miquel Garcia, Estefania Casana, Sergio Muñoz, Claudia Jambrina, Victor Sacristan, Sylvie Franckhauser, Ignasi Grass, Veronica Jimenez, Fatima Bosch

**Affiliations:** 1 Center of Animal Biotechnology and Gene Therapy, Universitat Autònoma de Barcelona, 08193 Bellaterra, Spain; 2 Department of Biochemistry and Molecular Biology, Universitat Autònoma de Barcelona, 08193 Bellaterra, Spain; 3 CIBER de Diabetes y Enfermedades Metabólicas Asociadas, Instituto de Salud Carlos III, Madrid, Spain

**Keywords:** Metabolism, Fat metabolism, Transcriptomics

## Abstract

Obesity and type 2 diabetes are two closely related diseases representing a serious threat worldwide. An increase in metabolic rate through enhancement of non-shivering thermogenesis in adipose tissue may represent a potential therapeutic strategy. Nevertheless, a better understanding of thermogenesis transcriptional regulation is needed to allow the development of new effective treatments. Here, we aimed to characterize the specific transcriptomic response of white and brown adipose tissues after thermogenic induction. Using cold exposure to induce thermogenesis in mice, we identified mRNAs and miRNAs that were differentially expressed in several adipose depots. In addition, integration of transcriptomic data in regulatory networks of miRNAs and transcription factors allowed the identification of key nodes likely controlling metabolism and immune response. Moreover, we identified the putative role of the transcription factor PU.1 in the regulation of PPARγ-mediated thermogenic response of subcutaneous white adipose tissue. Therefore, the present study provides new insights into the molecular mechanisms that regulate non-shivering thermogenesis.

## Introduction

Obesity is very strongly associated with insulin resistance and Type 2 Diabetes (T2D) and represents a serious health, social, and economic problem due to its high prevalence worldwide. Despite the clinical significance of T2D and obesity, no effective treatments are available and additionally, anti-obesity drugs and bariatric surgery are not devoid of significant side-effects and risks^1^. Thus, there is an urgent need for novel and safe approaches to prevent and combat the current T2D-obesity epidemic.

The induction of non-shivering thermogenesis in adipose tissue has been shown to increase energy expenditure, improve glucose metabolism and insulin action, and reduce body fat mass in both animal models and humans^2,3^. Non-shivering thermogenesis mainly occurs in brown adipose tissue (BAT), in which the uncoupling of oxidative phosphorylation, mediated by the presence of the uncoupling protein 1 (UCP1), results in heat production. Human individuals with active BAT detected by ^18^F-fluorodeoxyglucose positron emission tomography–computed tomography scans have significantly improved metabolic profiles and cardiometabolic health^4^. Upon appropriate stimuli, thermogenic brown-like adipocytes (termed beige or brite) can also arise within subcutaneous white adipose tissue (WAT), a process known as browning^5–7^. Beige adipocytes share several key characteristics with brown adipocytes, including the expression of UCP1 and the appearance of multilocular lipid droplets^5–7^. More than 50 transcriptional regulators control brown or beige adipocyte differentiation, most of them under the control of master regulators such as peroxisome proliferator-activated receptor-γ (PPARγ) and their partners CCAAT/enhancer-binding protein-ß (C/EBPβ), PR domain zinc-finger protein 16 (PRDM16) and PPARγ co-activator-1α (PGC1α)^8^.

Since human adults present adipose depots with functional thermogenesis, thermogenic activation could be an important target for the prevention and treatment of T2D and obesity^9–12^. In fact, stimulation of browning of WAT may represent a relevant approach to increase energy expenditure because of the larger presence of subcutaneous WAT compared to BAT in humans. BAT and browning of WAT are activated by cold exposure in both rodents and humans, and these processes are positively correlated to cold-induced increases in energy expenditure^13^. However, the transcriptomic networks regulating thermogenic induction in adipose tissue have not been fully elucidated.

In the present study, to characterize the regulatory network that can drive thermogenesis and unravel depot-specific responses, transcriptomic analysis of both mRNA and microRNA (miRNA) of interscapular brown (iBAT), inguinal (iWAT) and epididymal (eWAT) adipose tissues from mice exposed to cold was performed. This analysis allowed the description of a novel gene regulatory network characterized by the upregulation of metabolism-related genes and the downregulation of genes associated with the immune system. This network is putatively coordinated by the transcription factor PU.1, which interacts with PPARγ, an essential transcription factor for both white and brown adipocytes differentiation^14–16^.

## Results

### Analysis of gene expression profiles of adipose depots during induction of non-shivering thermogenesis

**Figure 1:**
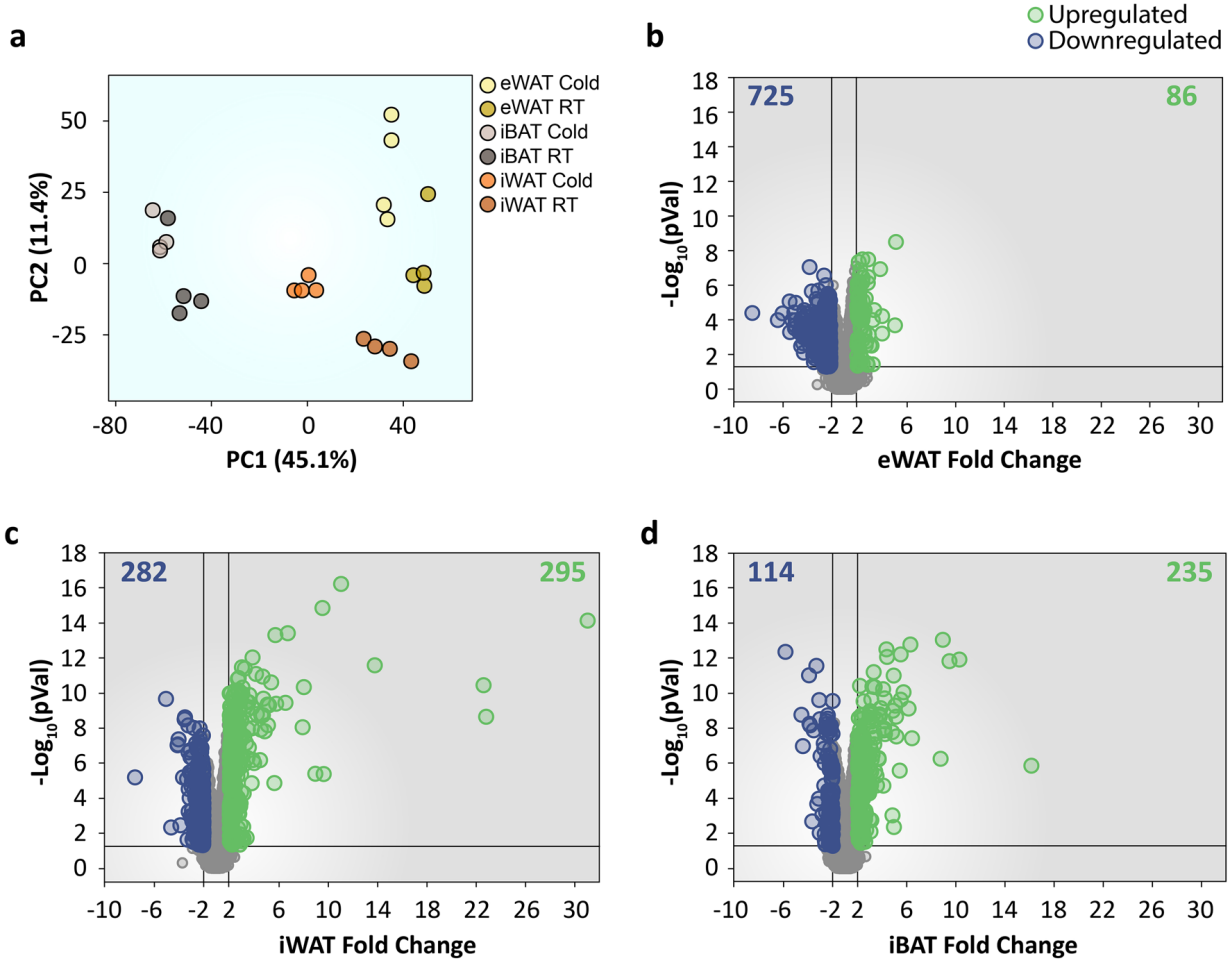
Gene expression analysis of different adipose tissue depots from mice exposed to cold. (**a**) Visualization of the data variance by principal component analysis. (**b-d**) Volcano plot displaying -log_10_ (p-value) vs. fold change of each gene expressed in (**b**) eWAT, (**c**) iWAT, and (**d**) iBAT. Green dots highlight significantly upregulated genes. Blue dots highlight significantly downregulated genes. The number of significant upregulated and downregulated genes are indicated in the top right and left corners, respectively. Data were obtained from two groups of mice that were either exposed to cold (4 °C) or maintained at room temperature (22 °C) for 4 days (n = 4/group).

To induce thermogenesis and the white-to-brown fat conversion, mice were exposed to cold (4 °C) for 4 days. Control mice remained at room temperature. Histological analysis of iWAT sections from cold-exposed mice showed the presence of numerous multilocular adipocytes compared with mice at room-temperature, indicating induction of browning (Supplementary Fig. 1a). This was parallel to increased expression of the thermogenic markers Ucp1 and Cidea in iWAT, further confirming enhancement of non-shivering thermogenesis (Supplementary Fig. 1b,c). Expression levels of mRNA in whole iBAT, iWAT and eWAT depots from mice exposed to cold or to room temperature were next evaluated using the microarrays Affymetrix Mouse Gene 2.1 ST. To gain insight into the association of the different samples, principal component analysis of the gene expression data was performed. The iBAT and the two WAT depots were separated mainly by the first component (PC1), whereas the second component (PC2) captured the tissue variability between iWAT and eWAT and, within each depot, the changes related to the cold exposure, thus samples exposed to cold have higher values (Fig. 1a). Accordingly, samples from cold-exposed mice grouped closer in PC1 with the samples from room temperature-exposed mice for the iBAT and eWAT than within the iWAT samples (Fig. 1a). Thereby, samples from iWAT showed decreased PC1, closer to iBAT, on cold exposed samples, indicating that cold-induced changes in the expression profile are more pronounced in iWAT than in the other depots (Fig. 1a).

To better understand the changes produced by cold in different adipose tissue depots, differential gene expression analysis was performed (Fig. 1b–d, Supplementary Tables S1 and S2). This analysis showed that eWAT from cold-exposed mice presented 725 downregulated and 86 upregulated genes in comparison with room temperature-housed mice (Fig. 1b, Supplementary Tables S1 and S2). In contrast, iWAT showed a lesser number of downregulated genes (282) and a higher number of upregulated genes (295) than eWAT (Fig. 1c, Supplementary Tables S1 and S2). The iBAT presented 114 genes that were downregulated and 235 that were upregulated (Fig. 1d, Supplementary Tables S1 and S2). Thus, these results point into the differential response of each adipose depot.

### Specific transcriptomic changes triggered by cold adaptation

**Figure 2:**
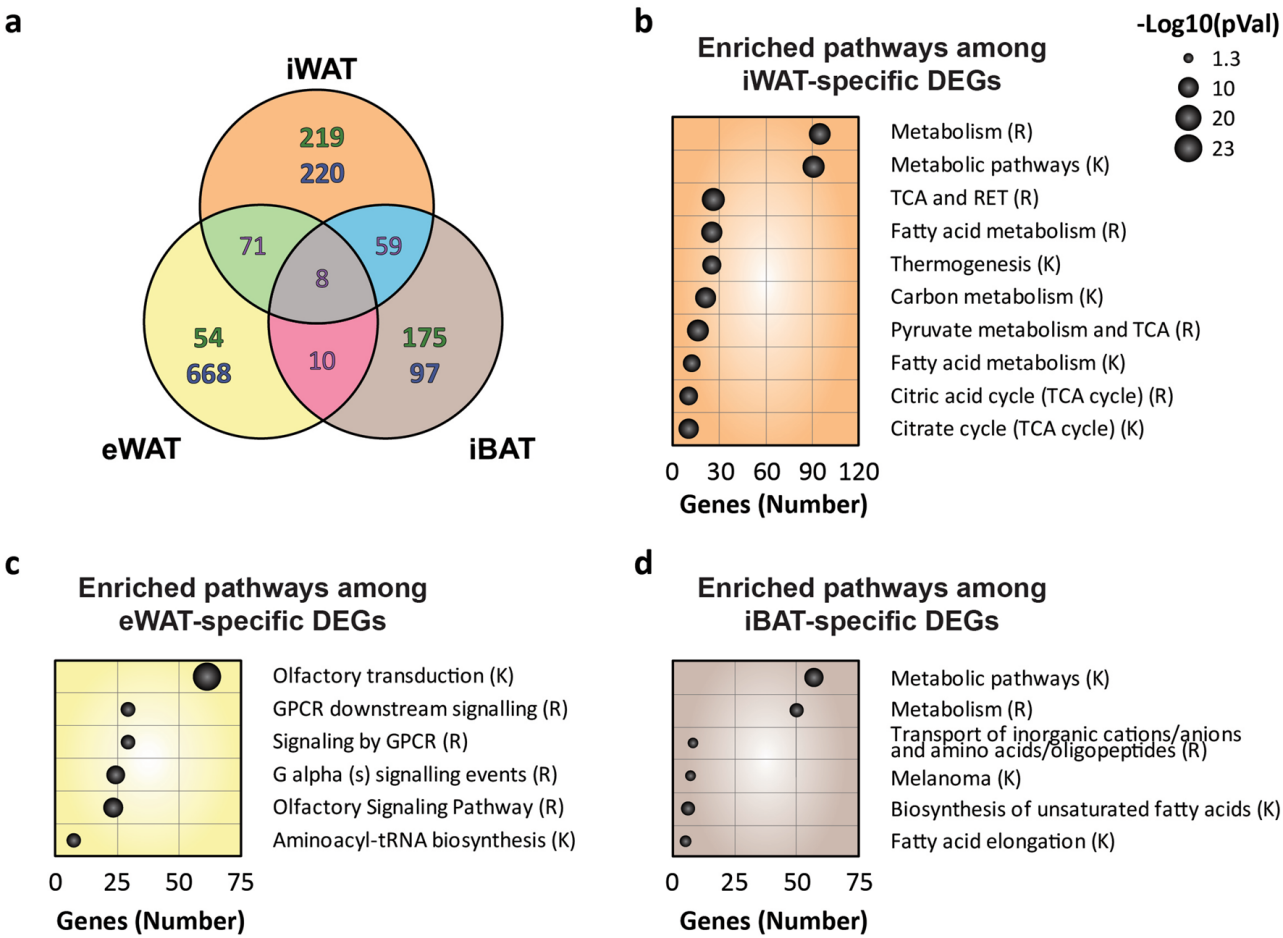
Functional analysis of depot-specific differentially expressed genes. (**a**) Set relationship analysis reporting the number of DEGs that were specific or common among the different adipose tissue depots. Highlighted in green: number of upregulated DEGs; Highlighted in blue: number of downregulated DEGs. (**b**) Ten most numerous enriched pathways among the iWAT-specific differentially expressed genes. (**c-d**) Pathways enriched among the (**c**) eWAT-specific and (**d**) iBAT-specific differentially expressed genes. -Log_10_ of the p-value correlates with the area of each ball, and the number of enriched genes of each pathway is indicated in the x-axis. *RET* respiratory electron transport. *TCA* tricarboxylic acid cycle. (R); Reactome. (K); KEGG Pathways. Data were obtained from two groups of mice that were either exposed to cold (4 °C) or maintained at room temperature (22 °C) for 4 days (n = 4/group).

To elucidate the specific effects of cold exposure on gene expression patterns in each depot, set relationships and pathway enrichment analyses were performed on differentially expressed genes (DEGs) (Fig. 2). Only 8 DEGs were shared between all three adipose depots (Supplementary Table S3). The iBAT and iWAT presented 59 DEGs in common, while iBAT and eWAT only shared 10 DEGs. Moreover, 71 genes were shared between both WAT depots. The specific DEGs of iWAT, eWAT, and iBAT were 439, 722, and 272, respectively (Fig. 2a).

Functional analysis using the Kyoto Encyclopedia of Genes and Genomes (KEGG) and Reactome databases revealed 45 enriched pathways among the iWAT specific DEGs (Fig. 2b, Supplementary Table S4). The 10 most enriched pathways in this tissue were related to metabolism (Fig. 2b). More specifically, an enrichment in different entries of the tricarboxylic acid cycle (TCA) pathway was observed, showing that this pathway is highly affected by cold exposure (Fig. 2b, Supplementary Table S4). TCA connects different metabolic pathways such as anaplerotic pathways through propionate metabolism or the degradation of lipids, both enriched in iWAT of cold-exposed mice (Fig. 2b, Supplementary Table S4). Moreover, several of the most upregulated genes in iWAT were also related to metabolism or thermogenesis (Supplementary Table S1). In contrast, genes downregulated in iWAT of cold-exposed mice were not related to these processes but were involved in pathways such as collagen formation and chemokine signalling (Supplementary Table S1). Functional analysis of eWAT did not show any enrichment in pathways related to metabolism or thermogenesis and only showed 6 different enriched pathways that were mainly related to the olfactory or G-protein signalling pathways (Fig. 2c). The iBAT also presented 6 enriched pathways, which were mainly related to lipid metabolism (Fig. 2d). This analysis clearly showed that transcriptomic responses to cold exposure differed markedly between eWAT and iWAT.

### Protein–protein interaction network analysis of DEGs

**Figure 3:**
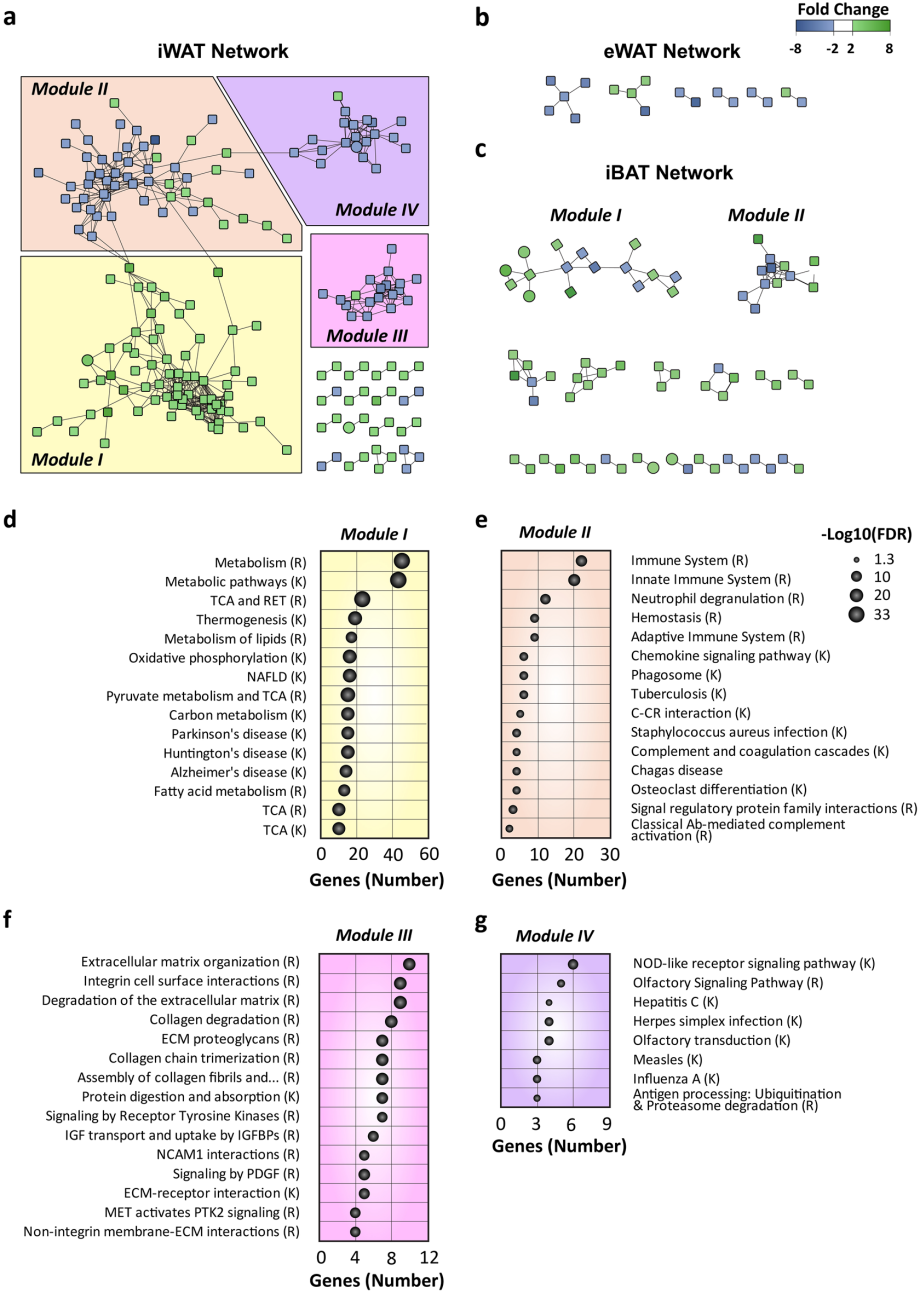
Protein‑protein interaction network of DEGs and module function identification. (**a**) The network of protein–protein interactions among the iWAT-specific differentially expressed genes, (**b**) among the eWAT-specific differentially expressed genes, and (**c**) among the iBAT-specific differentially expressed genes. (**d**–**f**) Fifteen most numerous enriched pathways among the encoding genes of the proteins located in (**d**) iWAT module I, (**e**) iWAT module II, and (**f**) iWAT module III. (**g**) Pathways enriched among the encoding genes of the proteins located in iWAT module IV. Green nodes indicate upregulated DEGs and blue nodes indicated downregulated DEGs. Edges stand for the regulatory association between any 2 nodes. Closer edges indicate stronger interaction validation. − Log_10_ of the p-value correlates with the area of each ball, and the number of enriched genes of each pathway is indicated in the x-axis. *RET* respiratory electron transport. *TCA* tricarboxylic acid cycle. *NAFLD* non-alcoholic fatty liver disease. *C-CR* cytokine-cytokine receptor, *Ab* Antibody. (R); Reactome. (K); KEGG Pathways.

To enhance the understanding of the molecular mechanisms underlying thermogenic activation of WAT, as well as their regulatory modules, protein–protein interaction (PPI) networks were constructed with the depot-specific DEGs. In iWAT, PPI network analysis allowed the identification of 374 nodes and 538 edges (Fig. 3a). This network was then organized by interaction strength, and 4 different clustered modules were observed and enumerated from module I to module IV (Fig. 3a, Supplementary Table S5). In sharp contrast, the specific DEGs of eWAT did not generate any network (Fig. 3b, Supplementary Table S5), while the PPI network analysis of specific DEGs of iBAT resulted in two small networks (Fig. 3c, Supplementary Table S5).

The main functions of the different modules of iWAT were then analysed by using pathway enrichment analysis. Genes related to module I were all upregulated, and presented enrichment in 74 different pathways, among which metabolic pathways were highly represented, especially pathways related to TCA, lipid metabolism, or thermogenesis (Fig. 3d, Supplementary Table S6). Module II was mainly composed of downregulated genes that displayed 40 pathways enriched mainly related to the immune system (Fig. 3e, Supplementary Table S6). Accordingly, the most downregulated genes of this module include C–C motif chemokine 8 (*Ccl8*), High-affinity immunoglobulin gamma Fc receptor I (*Fcgr1*), Lymphocyte antigen 86 (*Ly86*) or Macrophage mannose receptor 1 (*Mrc1*). Enriched pathways in module III were related to extracellular functions (Fig. 3f, Supplementary Table S6). Finally, module IV presented pathways related to pathogen response (Fig. 3g). In contrast, only 2 modules were identified in iBAT. In this depot, genes from module I were related to cancer pathways, while module II presented enriched pathways related to chemokine signalling (Supplementary Table S6). These results clearly demonstrated that the transcriptomic response underlying thermogenic activation was more extensive and interconnected in iWAT than in other adipose depots.

### Identification of transcription factors regulating iWAT network of DEGs

**Figure 4:**
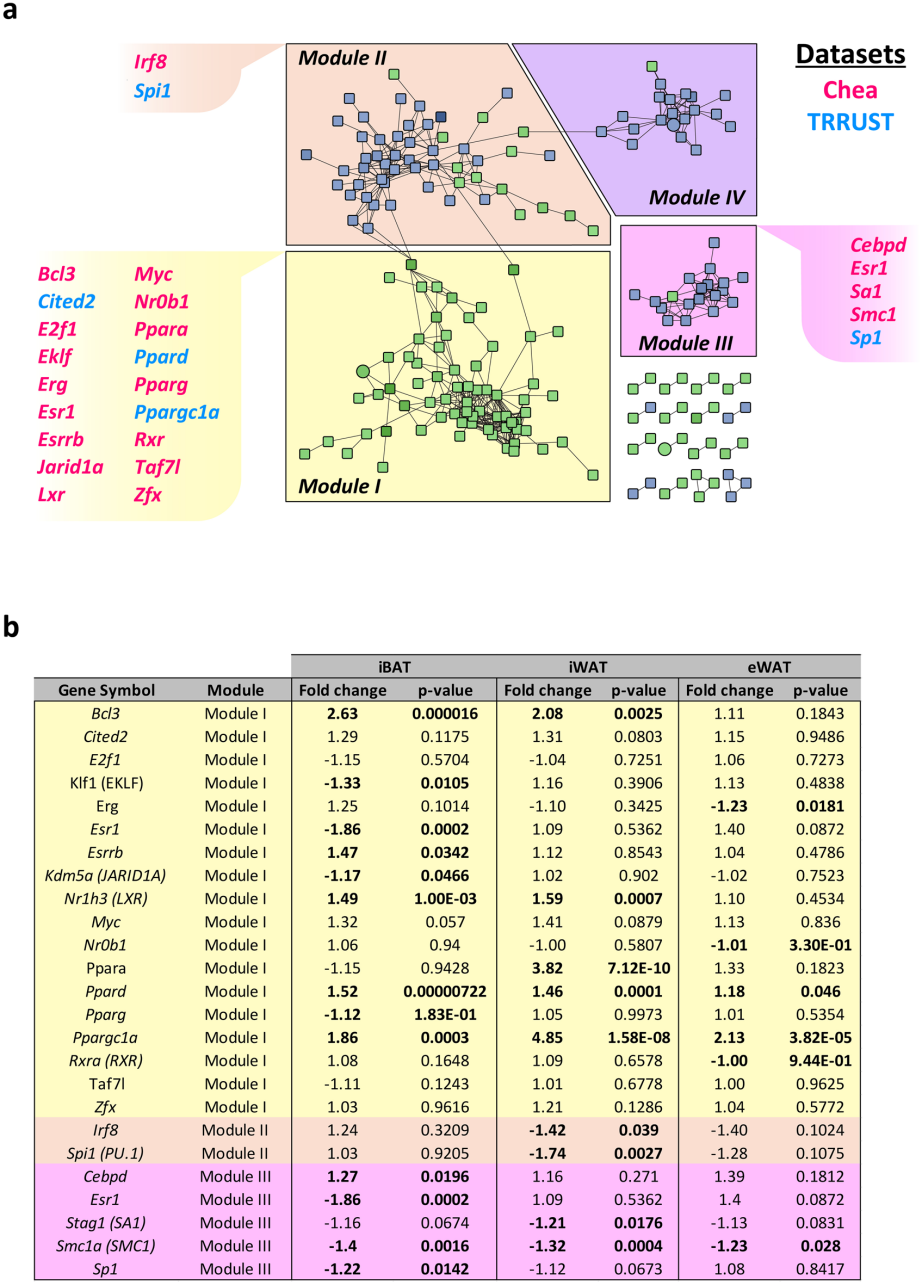
Transcription factors with a putative regulatory role in the iWAT-specific DEGs network. (**a**) Putative regulatory transcription factors determined by ChEA (pink) or TRRUST (light blue) are indicated for each iWAT module. (**b**) Fold change and p-values of the different selected transcription factors for each tissue. Factors with a significant differential expression were highlighted in bold.

Transcription factors putatively involved in transcriptional control of iWAT gene modules were then identified using the Enrichr application^17^ and the ChEA and TRRUST datasets^18,19^. Combining both libraries, the analysis of genes from module I showed 18 transcription factors as possible regulators of these genes, among which peroxisome proliferator activated receptor alpha (PPARα), peroxisome proliferator activated receptor gamma (PPARγ), peroxisome proliferator activated receptor delta (PPARδ), or PGC1α (*Ppargc1α,*) (Fig. 4a, Supplementary Table S7). The same approach with genes included in module II allowed the identification of the transcription factors interferon regulatory factor 8 (IRF8) and Spi-1 proto-oncogene (*Spi1*, PU.1) as candidates for the regulation of this module (Fig. 4a). In addition, while 5 transcription factors (CCAAT enhancer binding protein delta (CEBPD), oestrogen receptor 1 (ESR1), stromal antigen 1 (*Stag1*, SA1), structural maintenance of chromosomes 1A (*Smc1a*, SMC1), and Sp1 transcription factor (SP1)) were pointed out as putative regulators of module III genes, none was identified in module IV (Fig. 4a). The expression pattern of the identified transcription factors putatively regulating genes from modules I, II and III was then examined (Fig. 4b). In module I, PPARα was specifically upregulated in iWAT (Fig. 4b, Supplementary Table S8). Other transcription factors such as BCL3 transcription coactivator (BCL3), PPARδ, or nuclear receptor subfamily 1 group H member 3 (*Nr1h3*, LXRα) were similarly upregulated in both iBAT and iWAT, and therefore were probably not driving the differential response in both depots (Fig. 4b). PGC1α was upregulated in all three depots, while transcription factors such as PPARγ did not present changes in any adipose tissue (Fig. 4b, Supplementary Table S8). In module II, the expression of the transcription factors IRF8 and PU.1 was significantly downregulated specifically in iWAT (Fig. 4b, Supplementary Table S8). Finally, in module III, only the transcription factor SA1 presented a moderate specific downregulation in the iWAT (Fig. 4).

### Differentially expressed miRNAs

**Figure 5:**
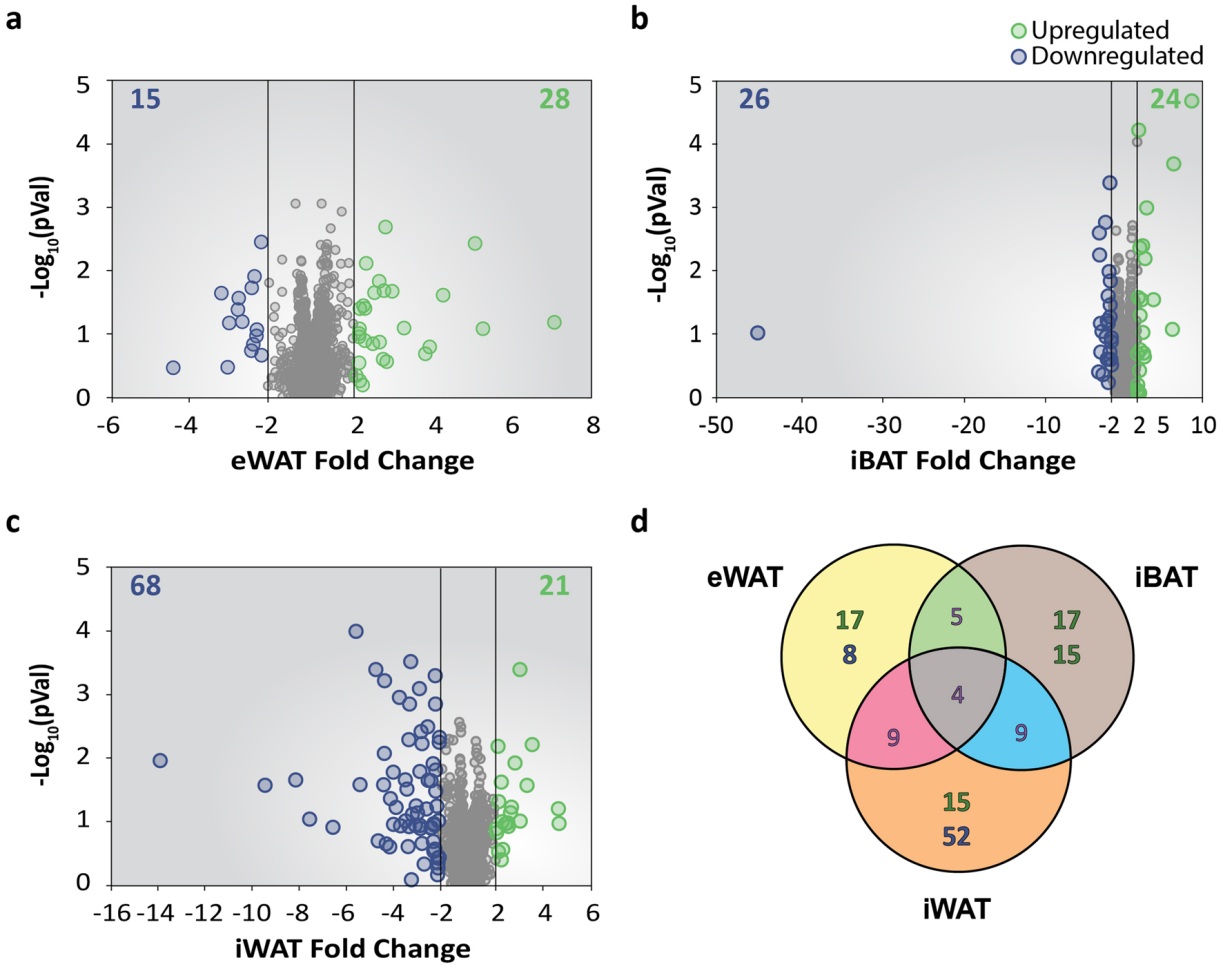
miRNA expression analysis of different adipose tissue depots from mice exposed to cold. (**a**–**c**) Volcano plot displaying -log_10_ (p-value) vs. fold change of each gene expressed in (**a**) eWAT, (**b**) iWAT, and (**c**) iBAT. Green dots highlight upregulated genes. Blue dots highlight downregulated genes. The number of upregulated and downregulated genes are indicated in the top right and left corners, respectively. (**d**) Set relationship analysis reporting the number of miRNAs that were specific or common among the different adipose tissue depots. Highlighted in green: number of upregulated genes; Highlighted in blue: number of downregulated genes.

Non-coding RNAs such as miRNAs have also been described to modulate gene expression. To analyse miRNA-target interactions (MTIs) potentially involved in transcriptomic changes, miRNA expression profiles were assessed from the same RNA samples obtained from adipose tissue depots of mice exposed to cold or room temperature. The eWAT depot presented 15 miRNAs downregulated and 28 miRNAs upregulated (Fig. 5a), while iBAT presented 26 and 24 miRNAs downregulated and upregulated, respectively (Fig. 5b). Finally, iWAT presented 68 downregulated miRNAs and 21 upregulated (Fig. 5c). From all these, the differentially expressed miRNAs specifically in eWAT were 25, whereas iBAT presented 32 of them (Fig. 5d). The iWAT was the depot that showed a higher number of differentially expressed miRNAs, presenting 15 upregulated and 53 downregulated miRNAs (Fig. 5d, Supplementary Table S9). Among these, several well-described regulators of the brown adipogenesis such as miRNA-27, miRNA-34a, miRNA-106b and miRNA-125-5p were identified^20–23^ (Supplementary Table S9).

### Gene regulatory network of iWAT

**Figure 6:**
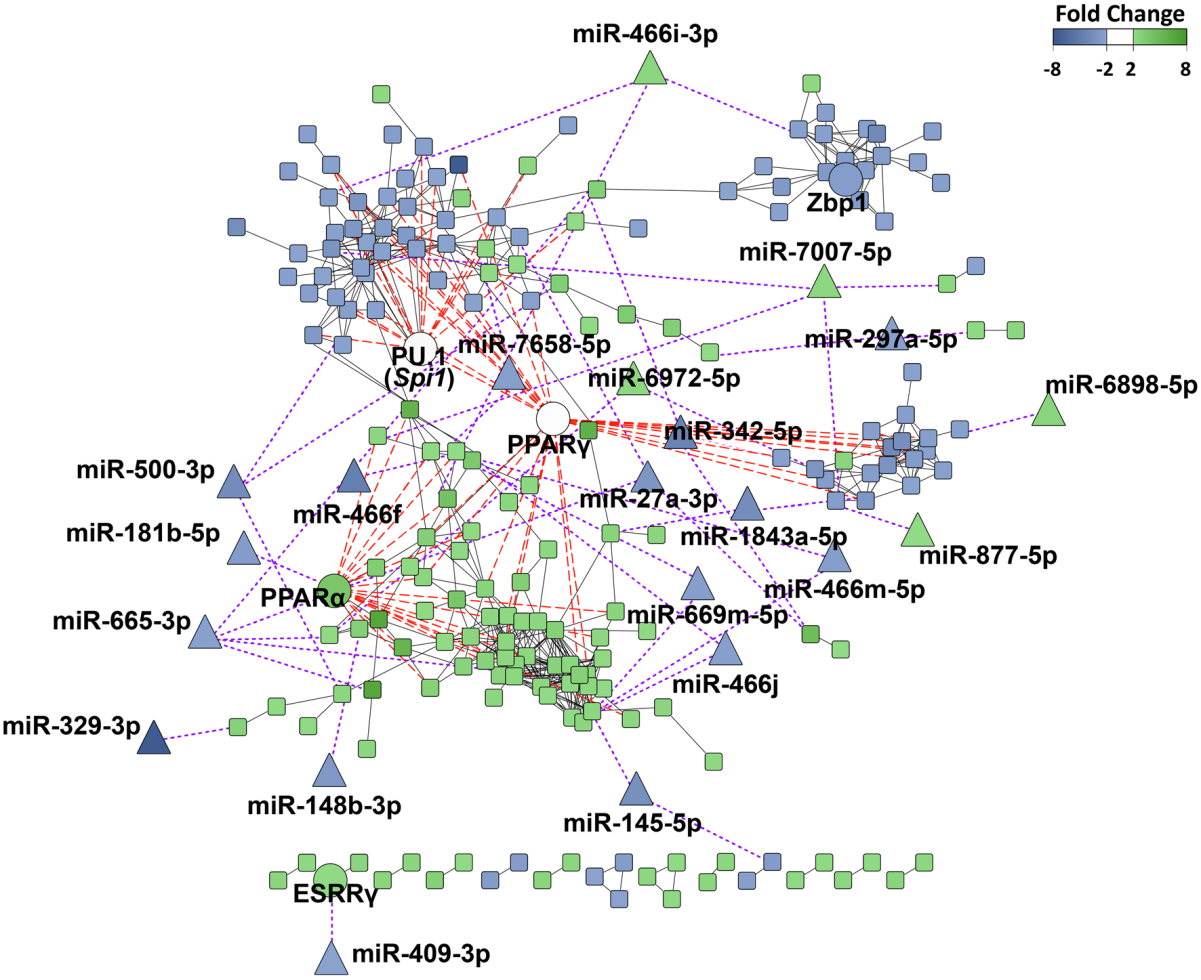
Regulatory network of the iWAT-specific differentially expressed genes. The regulatory network of the iWAT-specific DEGs was generated using the Cytoscape v3.9 software (https://cytoscape.org). Square nodes represent genes, circular nodes represent transcription factors and triangular nodes represent miRNAs. Long red dashed lines indicate interactions between transcription factors and genes. Short purple dashed lines indicate interactions between miRNAs and genes. Black lines indicate interactions between genes.

To further analyse the specific regulatory network in iWAT, the gene regulatory nodes comprising the identified transcription factors and miRNAs (Figs. 4 and 5) were integrated into the iWAT PPI network (Fig. 6). First, statistically significant interactions between transcription factors and their target genes were incorporated in the network (Fig. 6, Supplementary Table S7). The integration of the different transcription factors in the iWAT network together with their expression values suggested a potential role of PPARα and PU.1 in the regulation of modules I and II, respectively. Moreover, the analysis of this gene regulatory network also revealed a high number of genes interacting with PPARγ. Altogether, these interactions highlighted a potential central regulatory role of PPARα, PPARγ, and PU.1 in the specific iWAT network (Fig. 6). Afterwards, to identify putative interactions between specific miRNAs identified in iWAT and genes from the different modules of this network, the miRWalk database was used^24^. The results from this analysis were filtered selecting only the interactions comprising miRNAs that were downregulated, and genes upregulated, or vice versa, and then included in the network (Fig. 6, Supplementary Table S9). Using this approach, 21 miRNAs presenting 43 interactions with genes from the modules were identified (Fig. 6, Supplementary Table S10). Among them, miR-181b-5p and miR-665-3p targeted *Pparα*, and miR-466j, miR-466 m-5p, miR-669 m-5p, miR-665-3p, and miR-6972-5p targeted metabolic genes, such as acyl-CoA thioesterase 2 (*Acot2*) or solute carrier family 2 member 4 (*Slc2a4*, GLUT4) (Fig. 6, Supplementary Table S10). Altogether, these results provide a gene regulatory network underlying the specific thermogenic adaptation of subcutaneous WAT.

## Discussion

Enhancement of thermogenic response in adipose tissue has been established as a potential therapeutic approach for the treatment of obesity and diabetes. A better understanding of the depot-specific interplay between different regulatory layers (mRNA, miRNA, and transcription factors), resulting in a coordinated thermogenic response, is a crucial step for the future development of such therapeutic approaches. In this work, we provide for the first time an integrative transcriptomic analysis of mRNA and miRNA in different adipose tissue depots, allowing the identification of differentially expressed genes and specific regulatory networks in response to cold exposure. This study was performed in whole adipose tissue and not in isolated adipocytes to provide a more comprehensive view of the adipose tissue biology and to unravel responses of the entire tissue to environmental changes.

Our results revealed a strong transcriptomic response in iWAT in comparison with eWAT and iBAT, and a rich and meaningful interaction network between iWAT genes. In this specific gene network, 4 different clusters composing modules I to IV have been identified. Analysis of these modules showed that modules I and II presented a higher number of genes and level of regulation than modules III and IV. Our analysis showed that an important part of the genes included in module I are upregulated and that the enriched pathways in this module are related to lipid metabolism, more specifically to fatty acid metabolism and oxidation. Indeed, an increase in genes related to the electron transport chain is fully in agreement with an adaptation of WAT towards a greater capacity to uncouple fatty acid oxidation for heat production. Accordingly, expression of genes involved in the mitochondrial respiratory chain, such as *Ndufb8, Ndufab1, Ndufa8, Ndufa9, Ndufb6, Ndufs8,* encoding for subunits of Complex I, *Cox5a, Cox5b, Cox6b1, Cox10* or *Cox15* encoding for subunits of Cytochrome c oxidase (Complex IV), and *Cyc1*, *Uqcrfs1*, *Uqcr10* encoding for subunits of Complex III were upregulated. Moreover, expression of *Prkaa2* (5'-AMP-activated protein kinase catalytic subunit alpha-2) and *Slc25a20* (Carnitine-acylcarnitine translocase) encoding for proteins involved in regulatory steps of fatty acid oxidation was also increased in iWAT after cold exposure. All these results are consistent with previous findings showing induction of genes involved in mitochondrial metabolism in WAT after cold exposure^25,26^. This is also in accordance with a report showing that lipid metabolism proteins enrichment was higher than that of proteins involved in all other metabolite pathways in BAT throughout cold adaptation^27^. Lipolysis has traditionally been considered to be a major source for the intracellular production of free fatty acids as substrates for thermogenesis in brown and beige cells^28^. However, it has been reported that lipolysis may be dispensable for cold-induced thermogenesis in BAT, although it plays an essential role by fuelling thermogenesis during fasting^29,30^. Our data are in line with these findings since lipolysis was not among enriched pathways after cold exposure, neither between DEGs nor in module I, and suggest that in these conditions transcriptional changes of lipolysis may not play a crucial role in cold-induced browning adaptation of subcutaneous WAT.

Our results also pointed out a major downregulation of genes related to the innate immune system, most of them composing Module II. Among them, genes encoding for proteins involved in pathogen binding and antigen presentation such as CD180 molecule (*Cd180*), cathepsin S (*Ctss*), lymphocyte antigen 86 (*Ly86*) or CD209 molecule (*Cd209*) were downregulated. In addition, in module II of our network, a decreased expression of chemokines and chemokine receptors such as C–C motif chemokine ligand 7 (*Ccl7*), C–C motif chemokine ligand 8 (*Ccl8*), C–C motif chemokine receptor 2 (*Ccr2*) or C–C motif chemokine receptor 2 (*Ccr5*) has been observed, as well as a downregulation of C–C motif chemokine ligand 2 (*Ccl2*, MCP-1) expression, a proinflammatory macrophage M1-phenotype marker. This decrease in genes related to the immune system supports previous findings showing a reduction in pro-inflammatory markers during thermogenesis induction^31,32^. An anti-inflammatory state may also be achieved through the activation and recruitment of alternatively activated macrophages (M2 type). In agreement with this, previous reports showed increased expression levels of M2 marker genes and the recruitment of M2 macrophages in the WAT browning process^31–33^. Accordingly, we show that expression of the M2-phenotype marker mannose receptor C-type 1 (*Mrc1*, CD206) was upregulated in iWAT after cold exposure. Altogether, these results suggest that cold adaptation of WAT involves an interplay between metabolism and the immune system.

The integration of regulatory nodes such as transcription factors and miRNAs was also performed to reveal gene regulatory networks that could orchestrate gene expression changes. Previous integrative transcriptomic studies reported a key role of PPARα, PPARγ and PGC1α in thermogenic activation by being directly linked with *Ucp1* expression^34^. Accordingly, here we identified PPARα, PPARγ and PGC1α as putative central regulators of the network of metabolic and thermogenic related genes. Among these factors, PGC1α activates a number of nuclear receptors and transcription factors for transcriptional induction of UCP1 and other mitochondrial genes involved in mitochondrial oxidative metabolism, and its expression has been shown to be induced by cold-stimulated β3-adrenergic signalling in brown and beige adipocytes^35^. In agreement, our results indicated increased expression levels of this cofactor in all adipose depots after cold exposure, but with apparent higher levels in iWAT. However, in our study, the transcription factor with more interactions with thermogenic and metabolic genes was PPARγ, clearly showing that this factor would play a central role in thermogenic adaptation, although we did not observe changes in its expression levels in adipose depots after cold exposure. These results may suggest that its regulation is not at the transcriptional level. Interestingly, it has been described that PPARγ actions may be modulated by PU.1, which has also been identified as a putative central regulator of browning of subcutaneous WAT in our study, and which was downregulated specifically in iWAT. PU.1 has been described to affect the functionality of PPARγ and it has been suggested that the downregulation of *Spi1* would facilitate the binding of PPARγ to its target sites^36^. PU.1 deficiency in knockout mice led to modifications of DNA binding sites of PPARγ, the PPARγ cistrome, without affecting its expression levels^36^. It has been described that the PPARγ cistrome is different in eWAT, subcutaneous WAT, or BAT and that increased expression levels of PU.1 block the binding affinity of PPARγ in adipocytes^37,38^. All these results suggest that different expression levels of PU.1 between adipose depots may contribute to their heterogeneous metabolic and thermogenic responses. PU.1 is also able to orchestrate innate and adaptative immune cell fate^39,40^ and may also be a key factor in the interplay between the immune system and metabolism during browning of WAT^41^. Thus, PU.1 downregulation in iWAT could also be linked to the downregulation of genes related to the immune system in module II, while upregulating target genes of PPARγ related to thermogenesis and metabolism in module I. Accordingly, it has been recently reported that adipocyte-specific ablation of PU.1 in aging mice led to increased energy expenditure^42^. The transcriptional network analysis in iBAT and eWAT also showed that PU.1 suppressed inflammatory transcriptional programs in WAT and enhanced Ucp1 and Ppargc1a gene expression in BAT in aging mice^42^. Altogether, these results pointed out PU.1 as a novel key regulator of thermogenesis, in part by modulating the interplay between metabolism and the immune system.

The immune system and metabolism can also be regulated by miRNAs^20,43,44^. In our study, among the miRNAs that affect these pathways, miRNA-181a, which interacts with *Pparα* in iWAT network, was specifically downregulated in this tissue. In agreement with our results, miRNA-181a knockout mice displayed increased expression of genes associated with M2 macrophages polarization, suggesting that this miRNA may mediate the interplay between metabolism and immune system^45^. Moreover, another 20 miRNAs interacting with genes from the iWAT network were specifically regulated. Among them, miR-665-3p, whose expression was -2.24 in iWAT, target genes involved in metabolism such as *Slc2a4*, G protein subunit alpha o1 (*Gnao1*), protein kinase AMP-activated catalytic subunit alpha 2 (*Prkaa2*), oxoglutarate dehydrogenase (*Ogdh*)*,* and *Pparα*. Although no evidence of its functional role in thermogenesis has been reported, overexpression of rat miRNA-665, which presents 98.9% of homology with mouse miRNA-665-3p, has been described to reduce mitochondria complex functionality as well as to reduce cAMP signalling pathway^46^. All these results suggest that miRNA-665-3p may be a key factor in thermogenesis control.

Our results also pointed out major differences between BAT, eWAT and iWAT depots in their response to cold. In eWAT, it has not been possible to construct a relevant interaction network of the specific DEGs, probably because this depot is more resistant to thermogenesis induction^47^, and to express genes regulating this process^48,49^. However, we have been able to detect marked changes in gene expression in iWAT, providing key data for the mechanisms underlying iWAT browning adaptation. These results are of particular interest from a translational point of view since subcutaneous WAT represents a very large depot, easily accessible in humans, and it may provide greater possibilities of manipulation to increase thermogenesis than other adipose depots.

In summary, through mRNA and miRNA integrated transcriptomic analysis, this study revealed, a specific gene regulatory network in iWAT during non-shivering thermogenesis. The genes of this network were related to several processes, such as metabolism and thermogenesis as well as the innate immune system and extracellular organization. This study revealed for the first time that PU.1 may be a putative key player in cold adaptative response of subcutaneous WAT. A better understanding of key transcriptional events underpinning subcutaneous WAT browning, focusing on pathways and networks rather than pinpointing individual genes, is crucial for the development of therapeutic approaches aiming at enhancing energy expenditure and overcoming the detrimental effects associated with metabolic disorders, including obesity and type 2 diabetes.

## Methods

### Animal procedures

Male mice of the strain C57Bl/6JOlaHsd from Envigo of 8 weeks of age were randomly divided into two groups (n = 4) that were either exposed during 4 days to 4 °C in refrigerator room or to room temperature (22 °C). Sample size determination was based on previous experience with similar studies. In addition, we tested that the mean body weight was statistically not different for each experimental group. Each mouse was housed individually, and the bedding of the cage was removed to keep mice from nesting to help regulate body temperature. Day and night cycles were maintained with 12 h of light and 12 h of darkness. Mice were fed ad libitum with a standard diet (2018S Teklad Global Diets®, Envigo). Mice were anaesthetized by inhalational anesthetic isoflurane (IsoFlo, Abbott Laboratories) and euthanized by decapitation. Adipose tissue samples were obtained in the morning and were immediately frozen in liquid nitrogen and then stored at -80 °C. All experimental procedures were carried out and reported following the Federation of Laboratory Animal Science Associations (FELASA) and ARRIVE guidelines, and the ethical and legal requirements from Generalitat de Catalunya, Spain, and the EU directives. All experimental procedures were approved by the Ethics Committee for Animal and Human Experimentation of the Universitat Autònoma Barcelona (UAB).

### Microarray analysis of miRNA and mRNA

For the analysis of the mRNA expression, whole adipose tissue samples (including both adipocytes and stromal vascular fraction) were homogenized using QIAzol reagents. Rneasy Mini Kit was used to extract total mRNA. For the expression analysis of miRNAs, whole tissues were homogenized, and miRNAs were extracted using the mirVana miRNA Isolation Kit (Thermofisher) following the kit instructions. Samples were sent and processed by Progenika Biopharma. RNA integrity was determined with the Agilent 2100 Bioanalyzer. For the mRNA microarray, 300 ng of RNA was used to synthesize cDNA with the GeneChip WT Plus Reagent kit (Thermofisher) following the manufacturer’s instructions. cDNA was fragmented using UDG (Uracil DNA Glycosylase) and APE1 (Apurinic/apyrimidinic endonuclease 1) and labelled with terminal transferase using the WT Terminal Labeling kit. The GeneTitan Hybridization Wash and Stain kit for WT array plates were used following the manufacturer’s instructions. This kit contained a hybridization cocktail which included labelled, fragmented target and hybridization controls. Labelled samples were hybridized to Affymetrix GeneChip™ Mouse Gene 2.1 ST.

For the analysis of the miRNA expression, miRNAs were extracted using the mirVana kit. From 500 ng of each sample, a Poly(A)-tail was first bound to the total RNA with a PolyA Polymerase, and then 3DNA dendrimers were biotin-labelled, following the Affymetrix FlashTagBiotin HSR protocol. A mix of RNA Spike controls included in the kit was added to the starting RNA to verify that the processing had proceeded as expected. The kit GeneAtlas™ Hybridization, Wash, and Stain Kit for miRNA Array Strips were used following the protocol by Progenika Biopharma to hybridize the samples to the GeneChip™ miRNA 4.1. Arrays were scanned using the Affymetrix GeneTitan Platform. For both sets of microarrays, the Expression Console™ Software version 1.4.0.38 was used to perform quality control and to normalize the microarray data using Robust Multi-array Average algorithm. The microarray data of mRNAs was deposited in Array Express (E-MTAB-8965) and GEO (GSE148361). Similarly, the microarray data of miRNAs was also deposited in both databases, with the identifier E-MTAB-8966 for Array Express and GSE148845 for GEO.

The principal component analysis was generated using NetworkAnalyst 3.0^50^. After analyzing the variance captured by each principal component (PC) in a scree plot, it was observed that the PC to retain that capture enough variance were the first and the second variance. Therefore, plotting PC1 vs PC2 was adequate to capture enough variation.

### Differential expression analysis

The Transcriptome Analysis Console version 4.0.2.15 (Affimetrix) was used to perform statistical tests for differential gene expression. For the identification of DEG specifically expressed in a particular adipose depot or overlapped among them, an absolute FC higher than 2 and a p-value lower than 0.05 were considered as a cut-off criterion. For the identification of differentially expressed miRNAs, an absolute FC higher than 2 was considered as a cut-off criterion.

### Functional analysis

Pathway enrichment analysis were performed using Targetmine with the databases KEGG Pathway and Reactome^51–53^. The correction of Benjamini Hochberg method was applied to decrease the false discovery rate. The adjusted p-value cut-off was set to 0.05, considering lower values as statistically significant.

### Construction of protein–protein interaction (PPI) network

Cytoscape software (version 3.7.2) was used to construct the PPI network of the specific DEG of each depot by selecting high confidence interactions (confidence value of 0.7) using the stringApp plugin (version 1.5.0) that import STRING networks into Cytoscape^54,55^. Pathway enrichment of the genes from the different modules was assessed using the STRING plugin.

### MiRNA-target network construction

The miRWalk database 3.0 was used to select interactions in mice between tissue-specific differentially expressed mRNA and miRNAs by selecting the results presented by miRTarBase^56,57^. The genes of each module were uploaded to miRWalk to determine potential interactions with miRNAs. The fold change and p-values from the microarray were combined with the miRNAs list and the specifically differentially expressed miRNAs were selected.

### Identification of regulatory transcription factors

Enrichr application^17,58^ was used to test the libraries ChEA 2016^18^ and TRRUST Transcription Factors 2019^19^ that could link transcriptional machinery with the differential expressed genes of each module. ChEA is a dataset of transcription factors target genes identified from ChIP-chip, ChIP-seq, and other transcription factors binding site profiling studies^18^, while TRRUST manually curates sentence-based text mining to identify interactions described in small-scale experimental studies of transcriptional regulations^19,59^. Genes with a significative adjusted p-value lower than 0.05 from each library were selected as putative regulators.

### Gene expression quantification by real-time quantitative PCR (qRT–PCR)

One microgram of RNA was reverse-transcribed using the Transcriptor First Strand cDNA Synthesis kit (Roche). qRT–PCR was performed in a Lightcycler (Roche) using the Lightcycler 480 SyBr Green I Master Mix (Roche) and the following primers: Bmp8b: 5’AGACAGCCTTTCATGGTT 3’, 5AGCTGCTTCTTCTTCAGTGG’3’; Ccl8: 5’AAAGCTGAAGATCCCCCTTC3’, 5’TCTGGAAAACCACAGCTTCC3’; Col1a2: 5’GATGGCTGCTCCAAAAAGAG3’, 5’CAA TGT CCA GAG GTG CAA TG3’; Col3a1: 5’AGGATCTGTCCTTTGCGATG 3’, 5’TCTCCAAATGGGATCTCTGG3’; Cox7a1: 5’AAAACCGTGTGGCAGAGAAG3’, 5’TACAGGACGTTGTCCATTCC3’; Cox8b: 5’TTCACAGTCGTTCCCAAAGC3’, 5’CCATGAAGCCAACGACTATG3’; Cidea: 5’AAACCATGACCGAAGTAGCC3’, 5’AGGCCAGTTGTGATGACTAAGAC3’; Elovl3: 5’GGACTTAAGGCCCTTTTTGG3’, 5’CCAACAACGATGAGCAACAG3’; Fabp3: 5’TCCATGTGCGAAGTGGAAC3’, 5’AGGATGAGTTTCCCGTCAAC3’; Gbp10: 5’CAGCATGATCACCATCAACC3’, 5’TTGGGGAAGACTTTGCTCTG3’; Kng2: 5’TCAGCTTCTGAGTTGGTTTCC3’, 5’GCAGAGGAGCAGTGTAGTAATG3’; Pgc1a: 5’ATGAATGCAGCGGTCTTAGC3’, 5’GGACGTCTTTGTGGCTTTTG3’; Ppara: 5’AGATTCGGAAACTGCAGACC3’, 5’GTATGACAAAAGGCG GGTTG3’; Phospho1: 5’AGCTGGAGACCAACAGTTTC3’, 5’TCCCTAGATAGGCATCGTAGTC3’; Pu.1: 5’ATGGAAGGGTTTTCCCTCAC3’, 5’TGATCGCTATGGCTTTCTCC3’; Rplp0: 5’ACTGGTCTAGGACCCGAGAA3’, 5’TCCCACCTTGTCTCCAGTCT3’; Ucp1: 5’GGATTGGCCTCTACGACTCAG3’, 5TGTAGGCTGCCCAATGAACA’3’; Tacr2: 5’CCATGTTCGTCAGCATCTACTC3’, 5’CCAGCCAGATGACAGCAATAA3’. Data were normalized to Rplp0 expression. After normalization, relative expression was calculated using the 2(− ΔΔC(T)) method^60^. The GraphPad Prism 7 software was used for statistical analyses. Data were analysed by unpaired Student’s t-test. Differences were considered significant when P < 0.05.

### Histology

Tissues were fixed for 12–24 h in 10% formalin, embedded in paraffin and sectioned. Sections were stained with haematoxylin–eosin. Images were obtained with a Nikon Eclipse 90i microscope (Nikon).

## Supplementary Information

Supplementary Information 1.

Supplementary Information 2.

Supplementary Information 3.

## Data Availability

The datasets generated during and/or analysed during the current study are available in the GEO repository: GSE148361 (https://www.ncbi.nlm.nih.gov/geo/query/acc.cgi?acc=GSE148361) and GSE148845 (https://www.ncbi.nlm.nih.gov/geo/query/acc.cgi?acc=GSE148845).
