# Supplementary material for: Integrated gene expression profiles reveal a transcriptomic network underlying the thermogenic response in adipose tissue
Source: Sci Rep. 2023 May 4;13:7266. doi: 10.1038/s41598-023-33367-w (PMC10160086; doi:10.1038/s41598-023-33367-w)
Supplement: Supplementary file 1 — Supplementary Information 1. [file 41598_2023_33367_MOESM1_ESM.pdf]

Supplementary Information for

Integrated gene expression profiles reveal a transcriptomic network underlying the thermogenic response in adipose tissue

Jordi Rodó[1,2], Miquel Garcia[1,2,3], Estefania Casana[1,2,3], Sergio Muñoz[1,2,3], Claudia Jambrina[1,2,3], Victor Sacristan[1,2,3], Sylvie Franckhauser[1,3], Ignasi Grass[1,2,3], Veronica Jimenez[1,2,3*] and Fatima Bosch[1,2,3*]

[1]Center of Animal Biotechnology and Gene Therapy, and [2]Department of Biochemistry and Molecular Biology, Universitat Autònoma de Barcelona, 08193 Bellaterra, and [3]CIBER de Diabetes y Enfermedades Metabólicas Asociadas, Instituto de Salud Carlos III, Spain.

Supplementary Figure 1. Induction of browning in iWAT of mice exposed to cold. Mice were exposed either to cold (4ºC) or room temperature (22 ºC) for 4 days.

(a) Representative images of hematoxylin–eosin staining of iWAT sections of mice exposed to 4 ºC or room temperature for 4 days. Scale bars, 100 and 50 µm (insets).

(b-c) Quantification by qPCR of the expression of the non-shivering thermogenesis markers Ucp1 (b) and Cidea (c) in iWAT (n=4/group). Data are presented as mean ± SEM; *P<0.05, ***P<0.001. FC, Fold change. RT, room temperature

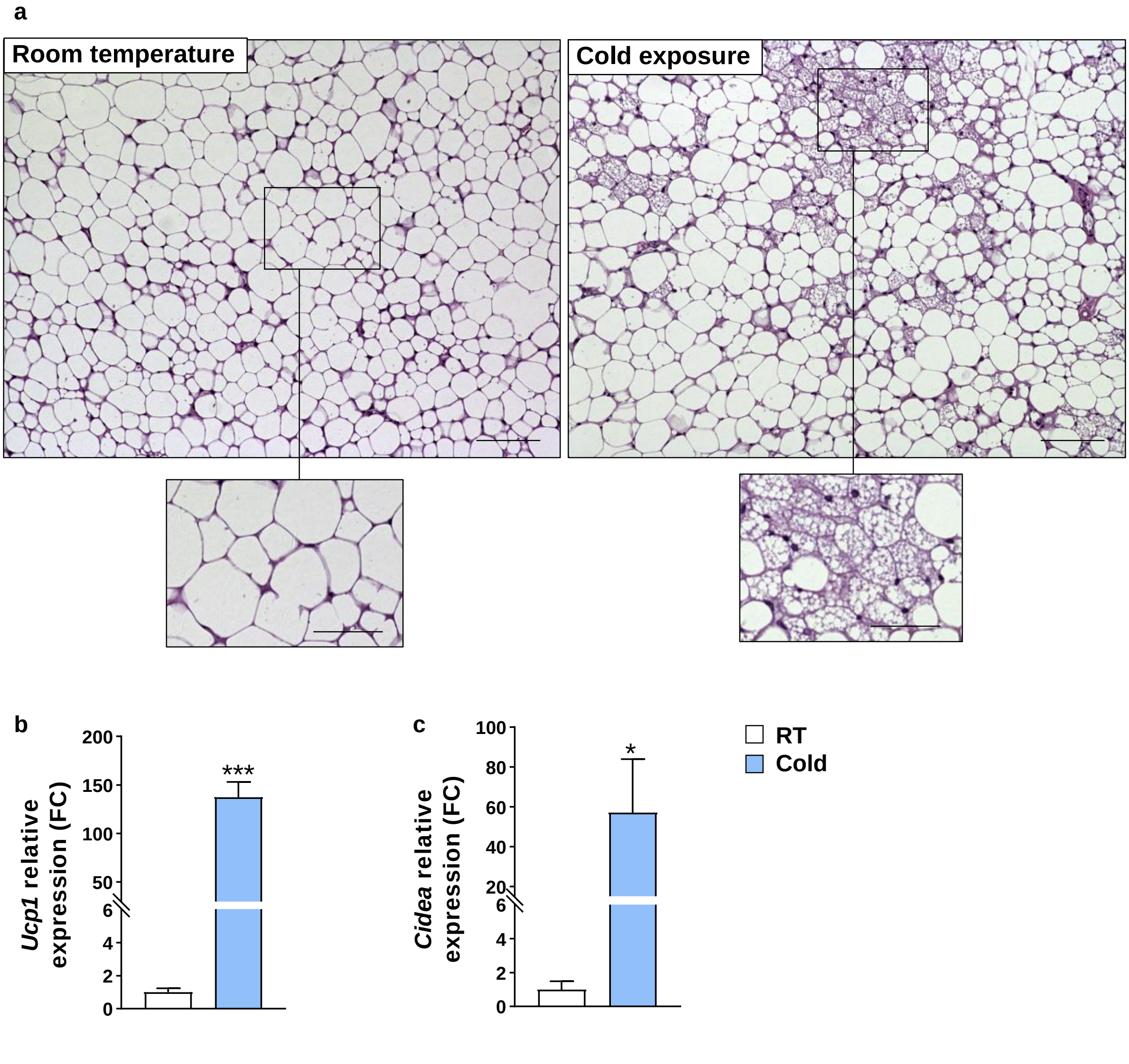

Supplementary Table S1. Most differentially expressed genes. Table indicating the most upregulated and downregulated genes in the iWAT, eWAT and iBAT depots. Expression levels together with fold change, p-value, and false discovery rate (FDR) values are shown. The most upregulated genes in each adipose depot are shown in green, while the most downregulated genes of the depot are indicated in blue. A * indicates that these probes can detect other nucleotides.

Excel file

Supplementary Table S2. Quantification by qPCR of the expression levels of DEGs in iBAT, iWAT and eWAT. Expression levels of several upregulated and downregulated genes in iBAT, iWAT and eWAT obtained by differential gene expression analysis of the microarray data were evaluated by qPCR (n=4/group for iWAT and eWAT and n=3/group for iBAT).

		**DEGs iWAT**	
	**Gene Symbol**	**Fold change**	**p-value**
Up regulated	*Fabp3*	30,91	1,15E-04
	Elovl3	101,61	5,00E-05
	Ucp1	137,34	1,30E-04
	Phospho1	11,52	3,47E-04
	Kng2	2,18	0,01086
	Cox7a1	17,57	2,00E-04
Down regulated	*Col1a2*	-3,97	1,81E-02
	Col3a1	-4,81	0,05925
	Ccl8	-8,78	0,02900
	Gbp10	-2,19	3,50E-02

		**DEGs eWAT**	
	**Gene Symbol**	**Fold change**	**p-value**
Up regulated	*Ucp1*	9,40	0,07078
	Cox8b	8,15	6,47E-07

		**DEGs iBAT**	
	**Gene Symbol**	**Fold change**	**p-value**
Up regulated	*Tacr2*	135,25	0,02716
	Bmp8b	5,22	0,01463

Supplementary Table S3. DEGs shared among iBAT, iWAT and iBAT.

DEGs shared among iBAT, iWAT and eWAT								
			iBAT		iWAT		eWAT	
ID	Gene Symbol	Description	Fold change	p-value	Fold change	p-value	Fold change	p-value
17255458	Phospho1; Zfp652	phosphatase, orphan 1; zinc finger protein 652	3,0	2,26E-09	11,0	6,29E-17	2,3	1,26E-07
17231571	B430219N15Rik	RIKEN cDNA B430219N15 gene	2,5	9,89E-06	5,1	4,77E-10	2,3	1,47E-05
17256388	Ttc25	tetratricopeptide repeat domain 25	2,5	0,0006	2,9	0,0003	2,0	0,01
17359678	Scd3	stearoyl-coenzyme A desaturase 3	6,4	3,47E-08	2,7	0,0007	2,4	0,0001
17277146	Acot4	acyl-CoA thioesterase 4	2,3	0,003	2,2	0,0003	2,5	3,26E-05
17230725	Mtarc1	mitochondrial amidoxime reducing component 1	2,2	1,60E-06	2,1	2,88E-06	2,0	3,08E-05
17433145	Rbp7	retinol binding protein 7, cellular	2,1	0,003	2,0	0,01	2,1	0,001
17456545	Lep	leptin	-2,6	0,0006	-2,1	0,006	-2,2	0,0001

Supplementary Table S4. Pathways enriched among the iWAT-specific differentially expressed genes. Table showing all the pathways enriched among the iWAT-specific differentially expressed genes together with the number of genes, the p-value, the -$\log_{10}$(p-Value), and the Reactome or KEGG pathway reference for each of them.

Pathway Enrichment	Genes	Pval	$-Log_{10}(pVal)$	Reference
The citric acid (TCA) cycle and respiratory electron transport	26	1,167E-12	11,93	R-MMU-1428517
Metabolic pathways	91	2,387E-11	10,62	mmu01100
Pyruvate metabolism and Citric Acid (TCA) cycle	16	7,471E-11	10,13	R-MMU-71406
Metabolism	95	1,172E-10	9,93	R-MMU-1430728
Fatty acid metabolism	25	6,959E-10	9,16	R-MMU-8978868
Carbon metabolism	21	1,508E-09	8,82	mmu01200
Citric acid cycle (TCA cycle)	10	1,070E-08	7,97	R-MMU-71403
Thermogenesis	25	4,341E-07	6,36	mmu04714
Citrate cycle (TCA cycle)	10	1,176E-06	5,93	mmu00020
Fatty acid metabolism	12	9,748E-06	5,01	mmu01212
Oxidative phosphorylation	17	1,271E-05	4,90	mmu00190
Non-alcoholic fatty liver disease (NAFLD)	18	1,284E-05	4,89	mmu04932
Parkinson disease	17	2,837E-05	4,55	mmu05012
Mitochondrial Fatty Acid Beta-Oxidation	9	3,856E-05	4,41	R-MMU-77289
Assembly of collagen fibrils and other multimeric structures	10	0,0001	3,88	R-MMU-2022090
Propanoate metabolism	8	0,0002	3,69	mmu00640
PPAR signaling pathway	12	0,0003	3,50	mmu03320
Huntington disease	18	0,0004	3,44	mmu05016
Fatty acid elongation	7	0,0008	3,09	mmu00062
Alzheimer disease	16	0,0012	2,91	mmu05010
Glyoxylate and dicarboxylate metabolism	7	0,0012	2,91	mmu00630
Biosynthesis of unsaturated fatty acids	7	0,0014	2,85	mmu01040
Glucagon signaling pathway	12	0,0015	2,82	mmu04922
Metabolism of lipids	34	0,0015	2,81	R-MMU-556833
AMPK signaling pathway	13	0,0021	2,67	mmu04152
Insulin resistance	12	0,0024	2,62	mmu04931
Collagen formation	10	0,0036	2,44	R-MMU-1474290
Respiratory electron transport, ATP synthesis by chemiosmotic	10	0,0036	2,44	R-MMU-163200
Glycolysis / Gluconeogenesis	9	0,0037	2,44	mmu00010
Collagen chain trimerization	7	0,0040	2,39	R-MMU-8948216
Pyruvate metabolism	6	0,0047	2,33	R-MMU-70268
Viral protein interaction with cytokine and cytokine receptor	11	0,0050	2,30	mmu04061
Respiratory electron transport	8	0,0077	2,11	R-MMU-611105
Aspartate and asparagine metabolism	4	0,0078	2,11	R-MMU-8963693
Chemokine receptors bind chemokines	8	0,0093	2,03	R-MMU-380108
Biosynthesis of amino acids	9	0,0100	2,00	mmu01230
Fatty acid degradation	7	0,0166	1,78	mmu00071
Cardiac muscle contraction	9	0,0219	1,66	mmu04260
Adipocytokine signaling pathway	8	0,0270	1,57	mmu04920
Regulation of pyruvate dehydrogenase (PDH) complex	4	0,0331	1,48	R-MMU-204174
Signaling by Retinoic Acid	6	0,0353	1,45	R-MMU-5362517
Peroxisomal lipid metabolism	5	0,0354	1,45	R-MMU-390918
Protein digestion and absorption	9	0,0373	1,43	mmu04974
Glyoxylate metabolism and glycine degradation	5	0,0381	1,42	R-MMU-389661
Collagen biosynthesis and modifying enzymes	7	0,0389	1,41	R-MMU-1650814

Supplementary Table S5. List of pathways enriched in the different modules of the different tissues.

iWAT Module I

Description	Genes	FDR value	-Log$_{10}$(FDR)	Reference
Metabolic pathways	43	6,020E-33	32,22	mmu01100
The citric acid (TCA) cycle and respiratory electron	23	3,890E-31	30,41	R-MMU-1428517
Metabolism	45	3,890E-31	30,41	R-MMU-1430728
Pyruvate metabolism and Citric Acid (TCA) cycle	15	4,570E-23	22,34	R-MMU-71406
Thermogenesis	19	3,820E-20	19,42	mmu04714
Oxidative phosphorylation	16	2,220E-19	18,65	mmu00190
Non-alcoholic fatty liver disease (NAFLD)	16	1,040E-18	17,98	mmu04932
Carbon metabolism	15	1,720E-18	17,76	mmu01200
Parkinson's disease	15	1,250E-17	16,90	mmu05012
Citric acid cycle (TCA cycle)	10	7,080E-17	16,15	R-MMU-71403
Citrate cycle (TCA cycle)	10	7,300E-16	15,14	mmu00020
Huntington's disease	15	7,300E-16	15,14	mmu05016
Alzheimer's disease	14	3,820E-15	14,42	mmu05010
Fatty acid metabolism	13	1,750E-12	11,76	R-MMU-8978868
Metabolism of lipids	17	1,320E-10	9,88	R-MMU-556833
Propanoate metabolism	7	1,990E-10	9,70	mmu00640
Respiratory electron transport, ATP synthesis by chemiosmotic coupling, and heat production by	8	3,740E-09	8,43	R-MMU-163200
Respiratory electron transport	7	1,260E-08	7,90	R-MMU-611105
Glycolysis / Gluconeogenesis	7	1,940E-08	7,71	mmu00010
Insulin resistance	8	1,960E-08	7,71	mmu04931
Mitochondrial Fatty Acid Beta-Oxidation	6	3,870E-08	7,41	R-MMU-77289
Cardiac muscle contraction	7	4,130E-08	7,38	mmu04260
AMPK signaling pathway	8	4,690E-08	7,33	mmu04152
Fatty acid metabolism	6	1,260E-07	6,90	mmu01212
Glucagon signaling pathway	7	2,030E-07	6,69	mmu04922
Complex I biogenesis	6	2,580E-07	6,59	R-MMU-6799198
Pyruvate metabolism	5	4,230E-07	6,37	R-MMU-70268
Glyoxylate metabolism and glycine degradation	5	4,560E-07	6,34	R-MMU-389661
Protein localization	6	9,480E-07	6,02	R-MMU-9609507
Pyruvate metabolism	5	1,030E-06	5,99	mmu00620
Import of palmitoyl-CoA into the mitochondrial	4	2,210E-06	5,66	R-MMU-200425
Regulation of pyruvate dehydrogenase (PDH)	4	2,570E-06	5,59	R-MMU-204174
Fatty acid degradation	5	3,430E-06	5,46	mmu00071
Signaling by Retinoic Acid	5	8,010E-06	5,10	R-MMU-5362517
Fatty acid elongation	4	9,100E-06	5,04	mmu00062
Peroxisomal protein import	5	1,120E-05	4,95	R-MMU-9033241
Adipocytokine signaling pathway	5	1,560E-05	4,81	mmu04920
beta-Alanine metabolism	4	1,740E-05	4,76	mmu00410
Biosynthesis of amino acids	5	1,820E-05	4,74	mmu01230
Mitochondrial fatty acid beta-oxidation of	3	2,460E-05	4,61	R-MMU-77286
Retrograde endocannabinoid signaling	6	2,660E-05	4,58	mmu04723
Lysine degradation	4	0,0001	3,85	mmu00310
2-Oxocarboxylic acid metabolism	3	0,0001	3,85	mmu01210
Insulin signaling pathway	5	0,0002	3,64	mmu04910
Glyoxylate and dicarboxylate metabolism	3	0,0004	3,42	mmu00630
Beta oxidation of palmitoyl-CoA to myristoyl-CoA	2	0,0004	3,36	R-MMU-77305
PPAR signaling pathway	4	0,0005	3,32	mmu03320

Peroxisomal lipid metabolism	3	0,0007	3,14	R-MMU-390918
Tyrosine metabolism	3	0,0008	3,10	mmu00350
Beta oxidation of decanoyl-CoA to octanoyl-CoA-	2	0,0011	2,96	R-MMU-77346
Tryptophan metabolism	3	0,0012	2,92	mmu00380
Valine, leucine and isoleucine degradation	3	0,0019	2,72	mmu00280
Heme biosynthesis	2	0,0025	2,60	R-MMU-189451
Lysine catabolism	2	0,0032	2,49	R-MMU-71064
Beta-oxidation of very long chain fatty acids	2	0,0035	2,46	R-MMU-390247
Phenylalanine metabolism	2	0,0059	2,23	mmu00360
Peroxisome	3	0,0059	2,23	mmu04146
Glucose metabolism	3	0,0063	2,20	R-MMU-70326
Histidine metabolism	2	0,0066	2,18	mmu00340
Butanoate metabolism	2	0,0079	2,10	mmu00650
Biosynthesis of unsaturated fatty acids	2	0,0079	2,10	mmu01040
Pentose phosphate pathway	2	0,0102	1,99	mmu00030
Galactose metabolism	2	0,0102	1,99	mmu00052
Fructose and mannose metabolism	2	0,0115	1,94	mmu00051
Energy dependent regulation of mTOR by LKB1-	2	0,0118	1,93	R-MMU-380972
Porphyrin and chlorophyll metabolism	2	0,0136	1,87	mmu00860
Cysteine and methionine metabolism	2	0,0181	1,74	mmu00270
Metabolism of carbohydrates	4	0,0217	1,66	R-MMU-71387
Sphingolipid de novo biosynthesis	2	0,0266	1,58	R-MMU-1660661
Central carbon metabolism in cancer	2	0,0323	1,49	mmu05230
Metabolism of xenobiotics by cytochrome P450	2	0,0325	1,49	mmu00980
Drug metabolism - cytochrome P450	2	0,0335	1,47	mmu00982
Glycolysis	2	0,0398	1,40	R-MMU-70171
RNA degradation	2	0,0452	1,34	mmu03018

iWAT Module II

Description	Genes	FDR value	$-Log_{10}(FDR)$	Reference
Innate Immune System	20	1,62E-12	11,79	R-MMU-168249
Immune System	22	2,18E-10	9,66	R-MMU-168256
Neutrophil degranulation	12	8,68E-08	7,06	R-MMU-6798695
Hemostasis	9	0,00011	3,96	R-MMU-109582
Signal regulatory protein family interactions	3	0,00015	3,82	R-MMU-391160
Chemokine signaling pathway	6	0,00031	3,51	mmu04062
Phagosome	6	0,00031	3,51	mmu04145
Staphylococcus aureus infection	4	0,00031	3,51	mmu05150
Tuberculosis	6	0,00031	3,51	mmu05152
Adaptive Immune System	9	0,00072	3,14	R-MMU-1280218
Complement and coagulation cascades	4	0,0012	2,92	mmu04610
Chagas disease (American trypanosomiasis)	4	0,0017	2,77	mmu05142
Osteoclast differentiation	4	0,003	2,52	mmu04380
Cytokine-cytokine receptor interaction	5	0,0042	2,38	mmu04060
Classical antibody-mediated complement	2	0,0056	2,25	R-MMU-173623
Platelet activation, signaling and aggregation	5	0,0064	2,19	R-MMU-76002
Pertussis	3	0,0081	2,09	mmu05133
Other semaphorin interactions	2	0,009	2,05	R-MMU-416700
IL-17 signaling pathway	3	0,013	1,89	mmu04657
Systemic lupus erythematosus	3	0,013	1,89	mmu05322
Role of phospholipids in phagocytosis	2	0,0158	1,80	R-MMU-2029485
Platelet calcium homeostasis	2	0,0167	1,78	R-MMU-418360
Natural killer cell mediated cytotoxicity	3	0,0187	1,73	mmu04650
Lysosome	3	0,0221	1,66	mmu04142
Prion diseases	2	0,0221	1,66	mmu05020
Cell surface interactions at the vascular wall	3	0,0223	1,65	R-MMU-202733
Apoptosis	3	0,0256	1,59	mmu04210
MHC class II antigen presentation	3	0,0276	1,56	R-MMU-2132295
Striated Muscle Contraction	2	0,0276	1,56	R-MMU-390522
Platelet degranulation	3	0,0289	1,54	R-MMU-114608
Rho GTPase cycle	3	0,0289	1,54	R-MMU-194840
DAP12 interactions	2	0,0289	1,54	R-MMU-2172127
Toll-like Receptor Cascades	3	0,0294	1,53	R-MMU-168898
Regulation of Complement cascade	2	0,0324	1,49	R-MMU-977606
Cross-presentation of soluble exogenous antigens	2	0,0369	1,43	R-MMU-1236978
ER to Golgi Anterograde Transport	3	0,0369	1,43	R-MMU-199977
Muscle contraction	3	0,0411	1,39	R-MMU-397014
Transcriptional misregulation in cancer	3	0,0426	1,37	mmu05202
Legionellosis	2	0,0442	1,35	mmu05134
G alpha (i) signalling events	4	0,0485	1,31	R-MMU-418594

iWAT Module III

Description	Genes	FDR value	$-Log_{10}(FDR)$	Reference
Integrin cell surface interactions	9	2,67E-16	15,57	R-MMU-216083
Collagen degradation	8	1,44E-14	13,84	R-MMU-1442490
Degradation of the extracellular matrix	9	1,44E-14	13,84	R-MMU-1474228
Extracellular matrix organization	10	4,92E-14	13,31	R-MMU-1474244
ECM proteoglycans	7	7,76E-14	13,11	R-MMU-3000178
Collagen chain trimerization	7	7,76E-14	13,11	R-MMU-8948216
Assembly of collagen fibrils and other multimeric	7	8,45E-13	12,07	R-MMU-2022090
Protein digestion and absorption	7	2,82E-11	10,55	mmu04974
NCAM1 interactions	5	4,39E-11	10,36	R-MMU-419037
Signaling by PDGF	5	3,92E-09	8,41	R-MMU-186797
Regulation of Insulin-like Growth Factor (IGF)	6	5,77E-09	8,24	R-MMU-381426
MET activates PTK2 signaling	4	4,14E-08	7,38	R-MMU-8874081
Signaling by Receptor Tyrosine Kinases	7	5,92E-08	7,23	R-MMU-9006934
ECM-receptor interaction	5	1,02E-07	6,99	mmu04512
Non-integrin membrane-ECM interactions	4	1,05E-07	6,98	R-MMU-3000171
Post-translational protein phosphorylation	5	1,61E-07	6,79	R-MMU-8957275
Axon guidance	6	1,74E-07	6,76	R-MMU-422475
Focal adhesion	5	4,68E-06	5,33	mmu04510
PI3K-Akt signaling pathway	5	0,0000472	4,33	mmu04151
Human papillomavirus infection	5	0,0000472	4,33	mmu05165
Proteoglycans in cancer	4	0,0000927	4,03	mmu05205
Platelet Adhesion to exposed collagen	2	0,00011	3,96	R-MMU-75892
GP1b-IX-V activation signalling	2	0,00013	3,89	R-MMU-430116
Anchoring fibril formation	2	0,00019	3,72	R-MMU-2214320
Signal Transduction	9	0,0002	3,70	R-MMU-162582
Crosslinking of collagen fibrils	2	0,00026	3,59	R-MMU-2243919
AGE-RAGE signaling pathway in diabetic	3	0,0003	3,52	mmu04933
Glycosaminoglycan metabolism	3	0,00031	3,51	R-MMU-1630316
Metabolism of proteins	7	0,00037	3,43	R-MMU-392499
Signaling by BMP	2	0,00053	3,28	R-MMU-201451
Keratan sulfate biosynthesis	2	0,00055	3,26	R-MMU-2022854
Relaxin signaling pathway	3	0,00056	3,25	mmu04926
Platelet Aggregation (Plug Formation)	2	0,00065	3,19	R-MMU-76009
Post-translational protein modification	6	0,00077	3,11	R-MMU-597592
GPVI-mediated activation cascade	2	0,00081	3,09	R-MMU-114604
Immunoregulatory interactions between a	2	0,0053	2,28	R-MMU-198933
Cell surface interactions at the vascular wall	2	0,006	2,22	R-MMU-202733
Amoebiasis	2	0,0096	2,02	mmu05146
Platelet activation	2	0,0116	1,94	mmu04611

iWAT Module IV

Description	Genes	FDR value	$-Log_{10}(FDR)$	Reference
NOD-like receptor signaling pathway	6	9,49E-08	7,02	mmu04621
Hepatitis C	4	4,46E-05	4,35	mmu05160
Herpes simplex infection	4	1,50E-04	3,82	mmu05168
Measles	3	8,30E-04	3,08	mmu05162
Influenza A	3	0,0013	2,89	mmu05164
Olfactory Signaling Pathway	5	0,0026	2,59	MMU-381753
Antigen processing: Ubiquitination & Proteasome degradation	3	0,018	1,74	MMU-983168
Olfactory transduction	4	0,0356	1,45	mmu04740

iBAT Module I

Description	Genes	FDR value	$-Log_{10}(FDR)$	Reference
Melanoma	5	1,58E-07	6,80	mmu05218
Breast cancer	5	2,54E-06	5,60	mmu05224
Glioma	4	5,40E-06	5,27	mmu05214
HDL remodeling	3	5,97E-06	5,22	MMU-8964058
Endocrine resistance	4	1,29E-05	4,89	mmu01522
Prostate cancer	4	1,29E-05	4,89	mmu05215
Bladder cancer	3	5,42E-05	4,27	mmu05219
Gastric cancer	4	5,42E-05	4,27	mmu05226
Ether lipid metabolism	3	5,84E-05	4,23	mmu00565
Hepatocellular carcinoma	4	5,90E-05	4,23	mmu05225
p53 signaling pathway	3	1,30E-04	3,89	mmu04115
Non-small cell lung cancer	3	1,30E-04	3,89	mmu05223
Cyclin D associated events in G1	3	1,30E-04	3,89	MMU-69231
Ras signaling pathway	4	1,40E-04	3,85	mmu04014
Pancreatic cancer	3	1,50E-04	3,82	mmu05212
Chronic myeloid leukemia	3	1,50E-04	3,82	mmu05220
Pathways in cancer	5	1,60E-04	3,80	mmu05200
HTLV-I infection	4	2,00E-04	3,70	mmu05166
Small cell lung cancer	3	2,20E-04	3,66	mmu05222
Cell cycle	3	4,60E-04	3,34	mmu04110
PI3K-Akt signaling pathway	4	4,70E-04	3,33	mmu04151
FoxO signaling pathway	3	5,20E-04	3,28	mmu04068
MicroRNAs in cancer	3	5,60E-04	3,25	mmu05206
Hepatitis B	3	6,00E-04	3,22	mmu05161
alpha-Linolenic acid metabolism	2	6,10E-04	3,21	mmu00592
Cushing's syndrome	3	7,00E-04	3,15	mmu04934
Cellular senescence	3	8,80E-04	3,06	mmu04218
Thyroid cancer	2	0,0011	2,96	mmu05216
Acyl chain remodelling of PI	2	0,0011	2,96	MMU-1482922
Kaposi's sarcoma-associated herpesvirus infection	3	0,0013	2,89	mmu05167
Proteoglycans in cancer	3	0,0013	2,89	mmu05205
Platelet degranulation	3	0,0014	2,85	MMU-114608
Acyl chain remodelling of PC	2	0,0014	2,85	MMU-1482788
Acyl chain remodelling of PS	2	0,0014	2,85	MMU-1482801
Acyl chain remodelling of PE	2	0,0014	2,85	MMU-1482839
Regulation of Insulin-like Growth Factor (IGF) transport and uptake by Insulin-like Growth Factor Binding Proteins (IGFBPs)	3	0,0014	2,85	MMU-381426
Synthesis, secretion, and deacylation of Ghrelin	2	0,0014	2,85	MMU-422085
Linoleic acid metabolism	2	0,0018	2,74	mmu00591
Binding and Uptake of Ligands by Scavenger	2	0,002	2,70	MMU-2173782
Endometrial cancer	2	0,0025	2,60	mmu05213
RMTs methylate histone arginines	2	0,0035	2,46	MMU-3214858
SCF(Skp2)-mediated degradation of p27/p21	2	0,005	2,30	MMU-187577
Transport of small molecules	4	0,0052	2,28	MMU-382551
Metabolism of lipids	4	0,0052	2,28	MMU-556833
Arachidonic acid metabolism	2	0,0054	2,27	mmu00590
Colorectal cancer	2	0,0054	2,27	mmu05210

Glycerophospholipid metabolism	2	0,0059	2,23	mmu00564
HIF-1 signaling pathway	2	0,0065	2,19	mmu04066
AMPK signaling pathway	2	0,0093	2,03	mmu04152
Oxytocin signaling pathway	2	0,0128	1,89	mmu04921
Post-translational protein phosphorylation	2	0,014	1,85	MMU-8957275
Jak-STAT signaling pathway	2	0,0145	1,84	mmu04630
Transcriptional misregulation in cancer	2	0,0151	1,82	mmu05202
Focal adhesion	2	0,0198	1,70	mmu04510
Viral carcinogenesis	2	0,02	1,70	mmu05203
Metabolism	5	0,0209	1,68	MMU-1430728
Transcriptional regulation by RUNX1	2	0,0209	1,68	MMU-8878171
Rap1 signaling pathway	2	0,0211	1,68	mmu04015
Thermogenesis	2	0,0237	1,63	mmu04714
Generic Transcription Pathway	3	0,0343	1,46	MMU-212436
SLC-mediated transmembrane transport	2	0,0343	1,46	MMU-425407
MAPK signaling pathway	2	0,0384	1,42	mmu04010
Human papillomavirus infection	2	0,0485	1,31	mmu05165

iBAT Module II

Description	Genes	FDR value	$-Log_{10}(FDR)$	Reference
Chemokine receptors bind chemokines	4	2,08E-07	6,68	MMU-380108
Class A/1 (Rhodopsin-like receptors)	5	4,12E-06	5,39	MMU-373076
G alpha (i) signalling events	5	7,24E-06	5,14	MMU-418594
Chemokine signaling pathway	4	4,31E-05	4,37	mmu04062
cAMP signaling pathway	3	0,0015	2,82	mmu04024
Cytokine-cytokine receptor interaction	3	0,0021	2,68	mmu04060
TNF signaling pathway	2	0,0079	2,10	mmu04668
Neutrophil degranulation	3	0,0082	2,09	MMU-6798695
NOD-like receptor signaling pathway	2	0,0141	1,85	mmu04621
Immune System	4	0,025	1,60	MMU-168256

Supplementary Table S6. List of genes represented in the PPI network of each tissue.

iWAT

Protein	Location	Array ID	Entrez ID	iBAT FC	iBAT pVal	iWAT FC	iWAT pVal	eWAT FC	eWAT pVal
Acaa2	Module I	17351811	52538	-1,22	**0,0332**	**2,66**	**1,67E-11**	1,43	**0,0006**
Acacb	Module I	17440826	100705	-1,07	0,9084	**2,03**	**1,07E-05**	1,95	**2,08E-05**
Acadvl	Module I	17265129	11370	1,16	0,1315	**3,03**	**1,14E-10**	1,66	**8,17E-05**
Aco2	Module I	17313394	11429	1,02	0,8652	**2,22**	**2,86E-09**	1,61	**1,42E-05**
Acot2	Module I	17277134	171210	1,83	**6,31E-05**	**3,53**	**3,43E-10**	1,63	**0,0058**
Acss1	Module I	17392690	68738	1,57	**0,0001**	**3,86**	**9,77E-13**	1,14	0,4546
Aldh3b2	Module I	17356028	621603; 734	1,69	**0,0002**	**2,11**	**5,85E-06**	1,84	**2,91E-05**
Aldh3b3	Module I	17356028	621603; 734	1,69	**0,0002**	**2,11**	**5,85E-06**	1,84	**2,91E-05**
Atpaf2	Module I	17263594	246782	1,42	**0,0122**	**2,48**	**1,21E-08**	1,59	**7,59E-05**
Cox10	Module I	17264107	70383	1,41	**0,0003**	**2,59**	**4,07E-09**	1,37	**0,0034**
Cox15	Module I	17364954	226139	1,52	**0,0009**	**2,36**	**2,52E-06**	1,35	**0,0316**
Cox5a	Module I	17517727	12858	1,2	0,0874	**2,92**	**1,31E-10**	1,32	**0,0015**
Cox5b	Module I	17290965	102641600	1,02	0,7859	**2,28**	**1,09E-05**	1,44	**0,0099**
Cox6b1	Module I	17489223	110323	1,03	0,6414	**2,08**	**3,25E-06**	1,59	**0,0002**
Cs	Module I	17238433	12974	1,04	0,213	**2,39**	**1,71E-08**	1,41	**0,001**
Cyc1	Module I	17312396	66445	1,09	0,2959	**2,84**	**7,74E-10**	1,72	**1,52E-05**
Decr2	Module I	17342581	26378	1,45	**0,0008**	**2,03**	**3,29E-07**	1,01	0,665
Dlat	Module I	17526861	235339	1,11	0,22	**2,44**	**1,15E-09**	1,96	**5,78E-07**
Dlst	Module I	17277352	78920	1,24	**0,0329**	**2,24**	**1,31E-09**	1,38	**0,0005**
Ecsit	Module I	17524914	26940	1,25	**0,0144**	**2,29**	**4,52E-07**	1,53	**0,0012**
Ehhadh	Module I	17329220	74147	1,78	**0,0002**	**4,21**	**1,65E-09**	1,83	**0,000069**
Etfdh	Module I	17406091	66841	1,01	0,5197	**2,46**	**7,58E-11**	1,37	**0,0028**
Fahd1	Module I	17342038	68636	1,5	**0,001**	**2,35**	**1,84E-08**	1,22	0,1742
Fam195a	Module I	17342483	68241	1,58	**0,0009**	**2,33**	**1,08E-08**	1,9	**4,96E-06**
Fmc1	Module I	17457430	66117	1,33	**0,0461**	**2,19**	**3,33E-07**	1,45	**0,0074**
Foxred1	Module I	17525363	235169	1,5	**0,0003**	**2,45**	**1,27E-08**	1,52	**0,0001**
Gys1	Module I	17477811	14936	1,25	**0,0104**	**2,09**	**3,03E-06**	1,38	**0,0024**
Hadha	Module I	17446643	97212	1,12	0,1067	**2,1**	**1,08E-10**	1,49	**1,49E-05**
Idh3a	Module I	17517390	67834	1,12	0,3725	**2,25**	**6,15E-08**	1,26	**0,0086**
Ldhb	Module I	17472517	16832	1,15	0,155	**3,05**	**5,16E-11**	1,42	**0,0028**
Lyrm5	Module I	17464238	67636	1,27	**0,0328**	**2,31**	**1,69E-08**	1,25	**0,0108**
Mdh2	Module I	17443310	17448	1,24	**0,0082**	**2,32**	**3,35E-10**	1,33	**0,0012**
Mecr	Module I	17419332	26922	1,19	0,0961	**2,13**	**2,28E-08**	1,33	**0,0011**
Ndufa8	Module I	17384525	68375	1,17	0,0913	**2,41**	**3,89E-09**	1,29	**0,003**
Ndufa9	Module I	17471166	66108	1,19	0,0743	**2,15**	**6,18E-09**	1,46	**0,0001**
Ndufab1	Module I	17495921	70316	1,21	0,0964	**2,24**	**8,51E-07**	1	0,162
Ndufb6	Module I	17424023	230075	1,13	0,2296	**2,02**	**1,94E-09**	1,34	**0,0003**
Ndufb8	Module I	17365134	67264	-1	0,667	**2,11**	**7,47E-07**	1,28	**0,0139**
Ndufs8	Module I	17361032	225887	1,03	0,7254	**2,37**	**2,61E-07**	1,5	**0,0017**
Ogdh	Module I	17247080	18293	1,08	0,1465	**2,7**	**7,31E-11**	1,33	**0,0053**
Pdhx	Module I	17388756	27402	1,22	0,058	**2,66**	**8,48E-09**	1,77	**6,4E-06**
Pdk2	Module I	17268120	18604	1,42	**0,0009**	**2,77**	**3,56E-08**	1,78	**3,12E-05**
Pdk4	Module I	17464654	27273	1,12	0,2893	**5,37**	**2,58E-11**	1,62	**0,0392**
Pfkl	Module I	17242376	18641	1,46	**0,0022**	**2,41**	**4,18E-07**	1,51	**0,0014**
Pfkm	Module I	17314577	18642	-1,09	0,7785	**2,3**	**0,0002**	1,6	**0,0208**
Pgk1	Module I	17537088	18655	1,36	**0,0016**	**2,31**	**1,46E-09**	1,41	**0,0001**

Ppara	Module I	17313862	19013	-1,15	0,9428	**3,82**	**7,12E-10**	1,33	0,1823
Ppif	Module I	17297750	105675	1,84	**0,0002**	**5,77**	**4,26E-10**	1,79	**0,006**
Ppp1r3b	Module I	17500543	244416	-1,03	0,9835	**2,78**	**0,0008**	1,07	0,6791
Prkaa2	Module I	17427746	108079	1,31	**0,0426**	**2,41**	**5,31E-05**	1,14	0,2263
Samm50	Module I	17313688	68653	1,11	0,2094	**2,54**	**2,39E-09**	1,29	**0,0276**
Sdhd	Module I	17526843	66925	-1	0,9942	**2,24**	**3,78E-09**	1,39	**0,002**
Slc25a20	Module I	17521875	57279	1,32	**0,0072**	**3,23**	**4,21E-12**	1,56	**7,51E-05**
Slc27a2	Module I	17375701	26458	1,33	0,3645	**5,63**	**1,41E-05**	1,24	0,8944
Slc2a4	Module I	17265096	20528	1,17	0,1253	**2,05**	**1,5E-07**	1,46	**0,0002**
Stradb	Module I	17213226	227154	1,15	**0,0265**	**2,14**	**3,39E-08**	1,25	**0,0108**
Sucla2	Module I	17301899	20916; 1052	1,14	0,141	**2,3**	**1,61E-10**	1,35	**0,0041**
Suclg1	Module I	17459716	56451	1,11	0,1965	**2,36**	**4,23E-09**	1,34	**0,006**
Tbrg4	Module I	17260416	21379	1,59	**0,0005**	**2,12**	**1,34E-06**	1,23	**0,016**
Timm44	Module I	17507262	21856	1,08	0,1604	**2,47**	**1,32E-10**	1,5	**2,93E-05**
Tomm40	Module I	17487395	53333	1,21	**0,0304**	**2,64**	**6,18E-09**	1,26	**0,0298**
Tysnd1	Module I	17233744	71767	1,85	**8,85E-07**	**2,12**	**1,18E-07**	1,63	**8,84E-05**
Ucp3	Module I	17480729	22229	1,42	**0,0033**	**2,94**	**3,55E-09**	1,75	**9,76E-05**
Uqcr10	Module I	17260023	66152	1,15	0,2163	**2,06**	**8,51E-08**	1,48	**0,0009**
Uqcrc1	Module I	17521996	22273	1,06	0,1335	**2,74**	**1,46E-11**	1,53	**1,66E-05**
Uqcrfs1	Module I	17291570	66694	1,44	**0,0166**	**2,26**	**9,24E-08**	1,99	**0,000018**
Zadh2	Module I	17352212	225791	1,13	0,0541	**2,33**	**6,6E-08**	1,38	**0,0022**
Actn2	Module II	17290603	11472	1,14	0,4266	**3,14**	**0,0203**	1,35	0,4116
Adrbk2	Module II	17451297	320129	1,62	**0,0001**	**2,45**	**1,73E-09**	-1,05	0,6354
Alox5ap	Module II	17444961	11690	-1,1	0,7558	**-2,28**	**0,000052**	-1,41	**0,0296**
Arhgap20	Module II	17517222	244867	-1,21	0,2413	**-2,17**	**0,003**	-1,19	0,1894
Atp2a1	Module II	17496211	11937	-1,59	0,7317	**3,02**	**0,0283**	1,02	0,9585
C1qa	Module II	17431619	12259	-1,02	0,9478	**-2,19**	**7,85E-05**	-1,2	0,2277
C1qb	Module II	17431607	12260	-1,22	0,3646	**-2,71**	**0,000011**	-1,21	0,135
Ccl2	Module II	17254041	20296	-1,12	0,5683	**-2,12**	**0,0039**	1,03	0,9984
Ccl7	Module II	17254047	20306	1,02	0,908	**-2,01**	**0,001**	-1,06	0,4193
Ccl8	Module II	17254065	20307	1,08	0,8456	**-7,56**	**6,69E-06**	-1,75	**0,0368**
Ccr2	Module II	17523650	12772	-1,13	0,9007	**-2,68**	**0,0001**	-1,54	**0,0121**
Ccr5	Module II	17523659	12774	-1,12	0,3193	**-2,55**	**0,0015**	-1,87	0,0799
Cd180	Module II	17289527	17079	-1,22	0,5108	**-2,28**	**0,0002**	-1,67	**0,0159**
Cd209a	Module II	17507161	170786	-1,32	0,3455	**-2,05**	**0,0004**	-1,63	**0,0047**
Cd48	Module II	17219397	12506	-1,24	0,439	**-2,18**	**0,0009**	-1,03	0,6075
Clec12a	Module II	17463509	232413	-1,08	0,6214	**-2,77**	**3,72E-05**	-1,95	**0,0019**
Clec4a3	Module II	17462738	73149	-1,03	0,7134	**-2,19**	**2,61E-05**	-1,3	0,2798
Ctsc	Module II	17480018	13032	-1,36	0,0998	**-2,14**	**2,71E-05**	-1,27	0,077
Ctsh	Module II	17520233	13036	1,01	0,8441	**-2,14**	**0,000046**	-1,19	0,7369
Ctss	Module II	17400375	13040	1,2	0,3779	**-2,25**	**0,0004**	-1,41	0,2667
Des	Module II	17214476	13346	-1,12	0,5961	**2,09**	**0,0022**	-1,11	0,5894
Emr1	Module II	17339013	13733	-1,11	0,9017	**-2,46**	**0,0002**	-1,53	0,0835
F13a1	Module II	17291881	74145	1,07	0,6443	**-2,16**	**1,68E-05**	-1,65	**0,0049**
Fcer1g	Module II	17229658	14127	-1,17	0,9839	**-2,3**	**0,0002**	-1,34	0,1044
Fcgr1	Module II	17408024	14129	-1,5	0,1696	**-3,21**	**3,08E-05**	-1,6	0,0611
Folr2	Module II	17493949	14276	-1,24	0,2989	**-3,67**	**6,74E-06**	-1,52	0,0542
Fyb	Module II	17309981	23880	1,15	0,4119	**-2,12**	**2,57E-05**	-1,67	**0,001**
Gnao1	Module II	17503884	14681	1,28	**0,0133**	**2,83**	**2,98E-10**	-1,23	**0,0315**
Hmha1	Module II	17235136	70719	-1,29	0,3144	**-2,08**	**0,0007**	-1,21	0,2012

Hpse	Module II	17450142	15442	1,33	**0,036**	**-2,17**	**6,08E-05**	-1,22	0,5824
Itgam	Module II	17483577	16409	1,09	0,765	**-2,11**	**0,0006**	-1,28	0,3509
Kdelr3	Module II	17313000	105785	-1,01	0,861	**-2,11**	**8,56E-05**	1,01	0,9037
Lair1	Module II	17485673	52855	1,01	0,4773	**-2,14**	**0,0002**	-1,49	**0,0258**
Ly86	Module II	17286587	17084	-1,44	0,1308	**-3,06**	**4,74E-07**	-1,81	**0,0012**
Lyz2	Module II	17245223	17105	-1,11	0,6495	**-2,08**	**4,74E-05**	-1,41	0,0945
Mmp3	Module II	17514515	17392	1,05	0,8578	**-2,43**	**1,84E-05**	1,08	0,4868
Mrc1	Module II	17367102	17533	-1,3	0,1871	**-2,94**	**0,000011**	-1,8	**0,0063**
Msr1	Module II	17508850	20288	-1,31	0,3497	**-2,24**	**0,0001**	-1,39	0,0622
Myot	Module II	17350267	58916	-1,44	0,6954	**3,38**	**0,0185**	-1,31	0,4163
Orm3	Module II	17414738	18407	1,41	**0,0401**	**2,09**	**2,57E-05**	-1,01	0,4658
P2rx5	Module II	17252609	94045	-1,28	**0,0487**	**2,21**	**1,11E-05**	1,9	**2,76E-05**
P2ry12	Module II	17405482	70839	-1,07	0,1679	**-2,25**	**0,0002**	-1,42	0,0524
Pirb	Module II	17485589	18733	1,15	0,4865	**-2,32**	**0,0097**	1,18	0,9809
Pld4	Module II	17279404	104759	-1,29	0,3928	**-2,45**	**0,0002**	-1,4	0,0553
Ppif	Module II	17297750	105675	1,84	**0,0002**	**5,77**	**4,26E-10**	1,79	**0,006**
Ptges2	Module II	17369821	96979	1,19	0,417	**2,22**	**4,89E-06**	1,56	**0,0023**
Ptprc	Module II	17227536	19264	1,01	0,9178	**-2,08**	**1,17E-06**	-1,58	**0,0049**
Rab1b	Module II	17361463	76308	1,38	**0,0023**	**2,02**	**2,12E-05**	1,25	0,09
Rhot2	Module II	17342414	214952	1,71	**3,75E-06**	**2,23**	**3,64E-09**	1,22	**0,007**
Serpina3i	Module II	17278296	628900	-1,24	0,1992	**2,02**	**0,0006**	1,05	0,7946
Sirpb1b	Module II	17404209	668101	1,08	0,5948	**-2,25**	**0,0003**	-1,3	0,1978
Srl	Module II	17327737	106393	1,16	0,6765	**2,01**	**0,0057**	1,01	0,8484
Thy1	Module II	17516462	21838	-1,05	0,6404	**-2,08**	**0,000084**	-1,14	0,4963
Tyrobp	Module II	17476364	22177	1,09	0,7641	**-2,14**	**0,002**	-1,11	0,5331
Aspn	Module III	17287160	66695	1,01	0,9776	**-2,17**	**1,4E-07**	-1,3	**0,0161**
B4galt6	Module III	17352985	56386	1,14	0,4514	**-2,24**	**0,0042**	-1,19	0,4519
Chrdl1	Module III	17545087	83453	-1,25	0,3459	**-2,43**	**1,24E-05**	-1,38	**0,0243**
Col1a1	Module III	17255260	12842	-1,68	**0,0014**	**-3,24**	**7,3E-09**	-1,67	**0,0017**
Col1a2	Module III	17455801	12843	-1,63	**0,0026**	**-3,54**	**3,3E-09**	-1,63	**0,0024**
Col5a1	Module III	17368569	12831	-1,1	0,1675	**-2,02**	**2,2E-06**	-1,28	0,0677
Col5a2	Module III	17222719	12832	-1,37	0,1407	**-2,07**	**2,61E-05**	-1,29	**0,0209**
Col6a1	Module III	17242177	12833	-1,13	0,1226	**-2,56**	**1,31E-07**	-1,57	**0,0006**
Col6a2	Module III	17242150	12834	-1,3	0,0961	**-2,2**	**7,02E-07**	-1,44	**0,0033**
Col6a3	Module III	17225413	12835	-1,08	0,487	**-2,42**	**1,05E-07**	-1,63	**0,0033**
Fbn1	Module III	17390879	14118	-1,02	0,9245	**-2,49**	**1,84E-07**	-1,3	0,1944
Fstl1	Module III	17325514	14314	-1,03	0,7714	**-2,95**	**3,04E-06**	-1,23	0,2876
Has2	Module III	17317167	15117	-1,15	0,2198	**-2,22**	**5,81E-05**	-1,64	**0,0076**
Igfbp3	Module III	17260474	16009	1,79	**0,0003**	**2,51**	**1,77E-07**	1,17	0,3887
Lum	Module III	17236811	17022	-1,44	**0,044**	**-4,09**	**8,85E-08**	-1,53	**0,0191**
Mmp2	Module III	17503825	17390	-1,63	**0,0049**	**-2,28**	**0,0002**	-1,46	0,0766
Postn	Module III	17397575	50706	1,05	0,8042	**-2,38**	**1,16E-07**	-1,11	0,5171
Rcn1	Module III	17389034	19672	-1,4	0,4289	**-2,44**	**0,0001**	-1,51	0,4993
Vcan	Module III	17294738	13003	-1,37	0,1015	**-3,05**	**1,87E-07**	-1,61	**0,0259**
Ddx60	Module IV	17501440	234311	-1,37	0,0988	**-2,36**	**0,0005**	-1,5	**0,0453**
Gbp5	Module IV	17403205	229898	-1,24	0,142	**-2,03**	**0,0003**	-1,47	**0,0393**
Gbp6	Module IV	17450501	626578	-1,48	**0,0247**	**-4,03**	**4,56E-08**	-1,41	0,0841
Gbp7	Module IV	17403224	229900	-1,82	**2,27E-05**	**-2,12**	**6,5E-07**	-1,5	**0,0011**
Gnal	Module IV	17351426	14680	-1,08	0,667	**-2,02**	**0,0019**	1,31	0,2304
Herc6	Module IV	17458962	67138	-1,42	**0,0053**	**-2,27**	**2,31E-07**	-1,54	**6,85E-05**

Ifi204	Module IV	17230045	15951	-1,17	0,2263	**-2,05**	**2,05E-05**	-1,69	**0,0126**
Ifit1	Module IV	17358832	15957	-1,22	0,4352	**-2,5**	**6,16E-06**	-1,36	**0,023**
Iigp1	Module IV	17350925	60440	-1,77	**7,06E-05**	**-2,31**	**4,12E-08**	-1,86	**0,000015**
Oas1a	Module IV	17452126	246730	-1,02	0,9737	**-2,3**	**0,0014**	-1,18	0,2882
Oas1g	Module IV	17452115	23960	1,66	**0,0313**	**-2,33**	**0,0072**	-1,42	0,3452
Oas2	Module IV	17452054	246728	-1,34	0,3183	**-2,65**	**0,0001**	-1,6	**0,0095**
Oasl2	Module IV	17441037	23962	1,08	0,55	**-2,48**	**1,81E-06**	-1,51	**0,0044**
Olfr294	Module IV	17493143	257904	1,05	0,8187	**-2,17**	**0,0076**	1,14	0,4182
Olfr376	Module IV	17252706	258924	-1,01	0,8817	**-2,75**	**0,0014**	-1,97	**0,0234**
Olfr765	Module IV	17246388	544748	-1,11	0,3185	**-2,03**	**0,0299**	-1,24	0,4616
Rnf126	Module IV	17242676	70294	1,83	**3,05E-05**	**2,19**	**1,03E-06**	1,26	0,0577
Rnf213	Module IV	17259078	672511	-1,37	**0,0092**	**-2,32**	**1,08E-08**	-1,43	**0,0004**
Rtp4	Module IV	17324446	67775	-1,59	**0,0004**	**-2,78**	**1,03E-08**	-1,63	**0,0001**
Zbp1	Module IV	17395079	58203	1,02	0,619	**-2,49**	**2,25E-06**	-1,4	**0,0166**
Adra1a	No module	17301502	11549	1,11	0,3602	**2,31**	**3,36E-08**	-1,06	0,5857
Apoo	No module	17536312	68316	-1,04	0,7955	**2,43**	**5,34E-05**	1,11	0,3972
Apoo-ps	No module	17296082	621156	1,04	0,5211	**2,5**	**2,61E-07**	1,63	**0,0006**
Aspa	No module	17265748	11484	1,36	**0,0031**	**2,1**	**7,39E-08**	1,53	**0,0002**
Ciapin1	No module	17511887	109006	1,44	**0,0156**	**2,44**	**2,5E-07**	1,5	**0,0234**
Cideb	No module	17306867	12684	1,25	0,5681	**2,83**	**0,0118**	-1,81	0,6
Cma1	No module	17306937	17228	-1,15	0,4026	**-2,11**	**0,0038**	-1,14	0,389
Coasy	No module	17256522	71743	1,47	**0,0031**	**2,03**	**3,29E-06**	1,94	**1,01E-05**
Cpa3	No module	17404337	12873	-1,16	0,215	**-2,57**	**1,67E-05**	-1,3	0,0942
Cyp1a1	No module	17517770	13076	1,4	0,0525	**2,37**	**1,02E-05**	1,57	**0,002**
Cyp2u1	No module	17410400	71519	1,67	**1,05E-06**	**2,47**	**7,67E-11**	1,62	**8,04E-06**
Esrrg	No module	17220627	26381	1,56	**0,0029**	**2,05**	**5,97E-06**	1,06	0,4542
Gfm1	No module	17398152	28030	1,47	**8,84E-05**	**2,06**	**1,59E-09**	1,38	**0,0002**
Glrx5	No module	17278345	73046	1,68	**1,92E-06**	**2,17**	**7,82E-10**	1,67	**9,62E-07**
Gpd1	No module	17314888	14555	1,49	**6,48E-05**	**2,37**	**9,04E-10**	1,91	**7,45E-08**
Gyk	No module	17543045	14933	1,73	**0,0386**	**9,63**	**4,32E-06**	**2,38**	0,1689
Lpar4	No module	17537112	78134	-1,18	0,3538	**-2,07**	**3,95E-05**	-1,4	0,0888
Mcpt4	No module	17306960	17227	-1,08	0,6086	**-2,08**	**0,0012**	-1,13	0,6613
Mrps23	No module	17254962	64656	1,2	0,0989	**2,14**	**7,07E-06**	1,54	**0,002**
Mrps27	No module	17289439	218506	1,31	**0,0163**	**2,3**	**3,48E-08**	1,36	**0,0077**
Ms4a6b	No module	17357688	69774	-1,17	0,6047	**-2,35**	**0,0002**	-1,24	0,1209
Ms4a6c	No module	17357671	73656	-1,13	0,8196	**-2,6**	**1,13E-05**	-1,38	**0,0409**
Myl6b	No module	17246154	216459	1,94	**0,0002**	**2,27**	**9,38E-07**	1,49	**0,0076**
Mylk2	No module	17377816	228785	-1,2	0,6647	**2,53**	**0,0124**	1,11	0,7321
Nat8l	No module	17436545	269642	1,09	0,5153	**2,15**	**1,92E-06**	1,83	**0,000013**
Pank1	No module	17364150	75735	1,53	**0,0129**	**4,7**	**1,9E-09**	1,64	**0,0113**
Ppargc1b	No module	17354739	170826	1,12	0,3044	**2,5**	**9,26E-07**	1,4	**0,02**
Sdr39u1	No module	17306929	654795	1,52	**0,0035**	**2,09**	**6,52E-08**	1,42	**0,0006**
Timm8a1	No module	17544486	30058	1,22	0,2985	**2,07**	**0,001**	1,39	0,0924
Timm9	No module	17281773	30056	1,15	0,5186	**2,26**	**2,61E-06**	1,39	**0,0094**
Xrcc3	No module	17284135	74335	1,22	**0,0256**	**2,12**	**2,53E-05**	1,3	0,0965
Xrcc6	No module	17313433	14375	1,32	0,0512	**2,34**	**8,39E-08**	1,35	**0,0093**

iBAT

Protein	Array ID	Entrez ID	iBAT FC	iBAT pVal	iWAT FC	iWAT pVal	eWAT FC	eWAT pVal
Abcg1	17335770	11307	**2,17**	**3,48E-06**	1,16	0,2934	1,45	**0,0055**
Acot1	17277140	26897	**2,59**	**0,0015**	1,79	**0,0133**	1,76	0,2389
Acot12	17289061	74156	**3,34**	**7,68E-08**	1,54	**0,0157**	-1,5	0,0535
Acot5	17277161	217698	**2,03**	**0,0007**	1,29	0,2189	1,01	0,6959
Acox2	17303412	93732	**2,12**	**1,78E-07**	1,19	0,3554	1,31	**0,0271**
Acsm3	17482310	20216	**-2,38**	**7,97E-05**	-1,14	0,4812	1,67	**0,0013**
Adamts1	17331705	11504	**2,29**	**1,42E-06**	1,29	0,0526	1,01	0,4468
Adamtsl3	17479769	269959	**-2,26**	**2,18E-06**	-1,07	0,2539	-1,01	0,7172
Alb	17438886	11657	**-3,68**	**0,002**	1,24	0,6634	-1	0,9184
Angpt1	17316780	11600	**-3,34**	**2,48E-12**	-1,53	**0,0008**	-1,06	0,4685
Apoe	17487381	11816	**-2,16**	**6,69E-09**	-1,14	0,1417	1,02	0,5216
Atf3	17231033	11910	**2,52**	**1,96E-06**	1,07	0,4736	1,3	0,6159
Cars2	17507440	71941	**2,47**	**2,65E-10**	1,16	0,218	1,03	0,9079
Ccnd1	17498502	12443	**2,57**	**8,98E-07**	-1,76	**0,005**	-1,05	0,8162
Cd274	17358544	60533	**-3**	**3,61E-07**	-1,1	0,5495	-1,52	0,0578
Cdkn1a	17335467	12575	**2,28**	**0,005**	1,97	**0,0067**	1,27	0,5898
Cfd	17235018	11537	**-2,23**	**2,27E-08**	-1,2	0,0821	-1,07	0,585
Chac1	17374807	69065	**2,2**	**3,35E-05**	1,25	**0,0194**	1,27	**0,0138**
Cpm	17237547	70574	**2,2**	**2,85E-06**	1,3	0,0519	1,43	**0,0019**
Cry1	17243644	12952	**2,33**	**2,92E-06**	1,58	**0,0096**	1,2	0,3507
Ctsz	17395155	64138	**3,37**	**4,06E-11**	1,13	0,1354	-1,07	0,529
Cx3cl1	17504130	20312	**2,4**	**4,41E-06**	-1,12	0,6094	1,26	0,1585
Cxcl13	17439367	55985	**2,07**	**0,0223**	-1,25	0,3676	1,94	0,3176
Cxcl3	17438975	330122	**-2,11**	**0,0336**	1,58	0,1041	-1,29	0,5107
Cxcl9	17449710	17329	**-2,42**	**2,63E-09**	-1,76	**3,69E-06**	-1,31	**0,0164**
Dusp6	17236882	67603	**2,09**	**5,12E-05**	1,23	0,1529	-1,11	0,265
E2f2	17420024	242705	**3,86**	**6,55E-10**	1,97	**4,77E-05**	1,24	0,4326
Etv6	17463673	14011	**2,17**	**5,26E-06**	1,31	**0,0333**	1,04	0,9139
F2r	17295130	14062	**2,01**	**1,15E-07**	1,06	0,3725	-1,03	0,7931
Fbp2	17293547	14120	**2,62**	**0,0005**	1,94	**0,0297**	1	0,8438
Ffar4	17359113	107221	**2,63**	**1,84E-07**	1,37	**0,0319**	1,51	**0,0077**
Fgf21	17490899	56636	**5,11**	**2,03E-09**	1,48	**0,0141**	1,03	0,8287
Fgf9	17300950	14180	**2,17**	**5,52E-06**	-1,03	0,7641	1,29	0,0712
Gbp2	17403268	14469	**-2,24**	**1,32E-08**	-1,59	**4,73E-05**	-1,26	**0,0059**
Gpx3	17249787	14778	**-2,17**	**9,16E-07**	-1,26	**0,0246**	-1,01	0,965
Grn	17256959	14824	**2,13**	**3,69E-07**	1,08	0,8494	1,16	0,3913
Gstt2	17241954	14872	**-2,01**	**2,12E-06**	1,34	**0,0123**	1,25	**0,0166**
Hcar1	17452709	243270	**-4,44**	**9,79E-08**	-1,33	0,3105	1,18	0,2028
Hcar2	17452705	80885	**-2,74**	**6,86E-08**	-1,42	**0,0074**	1,58	**0,0044**
Hist1h4h	17285815	69386	**2,38**	**0,0031**	-1,24	0,3205	-1,18	0,2466
Hist1h4i	17291053	319158	**2,24**	**4,7E-07**	1,33	**0,0358**	1,15	0,1164
Hist1h4n	17291019	319161	**2,41**	**0,0022**	-1,14	0,2351	-1,09	0,6894
Igf1	17236288	16000	**-2,38**	**9,75E-09**	-1,82	**7,69E-06**	-1,52	**0,001**
Impdh1	17465373	23917	**-2,22**	**0,0001**	1,02	0,3343	1,41	0,0748
Klk1b11	17477314	16613	**2,26**	**0,0097**	-1,06	0,5517	1,13	0,714
Krt17	17269415	16667	**3,1**	**0,0017**	1,32	0,3101	1,22	0,839

Krt36	17269343	16673	**2,15**	**0,0008**	1,26	0,3342	1,01	0,905
Mlycd	17506081	71839; 566	**2,43**	**1,27E-05**	1,07	0,5193	1,04	0,3445
Nfil3	17292634	18030	**2,02**	**1,77E-06**	1,02	0,848	-1,17	0,6218
Ntrk3	17492244	18213	**-3,11**	**2,26E-10**	-1,78	**2,16E-05**	1,01	0,7551
Oscar	17485520	232790	**3,14**	**5,56E-07**	1,17	0,1137	1,32	0,2813
Pik3r1	17295796	18708	**-2,29**	**4,7E-07**	-1,43	**0,005**	-1,63	**0,0019**
Pla2g12a	17402588	66350	**2,28**	**2,49E-08**	1,88	**7,75E-08**	1,45	**0,0009**
Pla2g16	17357072	225845	**-2,03**	**1,94E-08**	-1,06	0,4704	1,01	0,8418
Pla2g7	17337796	27226	**2,08**	**1,15E-05**	1,29	**0,0211**	1,19	0,3781
Pon1	17464614	18979	**-2,04**	**0,0011**	-1,42	0,098	1,13	0,4087
Ptgds	17382592	19215	**4,94**	**0,0042**	1,09	0,8717	1,57	**0,0292**
Ptger3	17403866	19218	**-2,46**	**3,51E-07**	-1	0,951	1,56	**0,0001**
Slc1a1	17358466	20510	**2,17**	**0,0129**	1,18	0,4924	-1,01	0,0752
Slc1a3	17315860	20512	**-2,22**	**8,98E-09**	-1,45	**0,001**	-1,04	0,4948
Slc38a2	17320928	67760	**2,15**	**1,17E-06**	1,19	0,1148	1,33	**0,0375**
Slc3a2	17362453	17254	**3,29**	**5,66E-12**	1,33	**0,0077**	1,05	0,5258
Smarcd1	17314872	83797	**3,1**	**2,07E-10**	1,4	**0,002**	1,06	0,6419
Spry4	17353957	24066	**2,67**	**2,93E-05**	1,37	0,0679	-1,26	0,2811
Tacr2	17233775	21337	**6,28**	**1,49E-13**	1,29	0,0611	-1,03	0,9344
Tectb	17360444	21684	**2,19**	**3,11E-06**	1,06	0,5272	1,12	0,3536
Trib3	17392925	228775	**3,69**	**4,73E-07**	1,54	**0,0097**	1,13	0,3122
Uck2	17229389	80914	**2,76**	**4,74E-05**	1,31	0,3086	1,6	**0,0131**
Ugt2b37	17449355	112417	**-2,22**	**9,05E-05**	-1,11	0,9531	-1,02	0,7655
Upp1	17247225	22271	**2,36**	**1,93E-07**	1,16	0,2684	1,11	0,3555
Yars	17418952	107271	**2,18**	**9,89E-07**	1,41	**0,0006**	1,28	**0,0223**

eWAT

Protein	Array ID	Entrez ID	iBAT FC	iBAT pVal	iWAT FC	iWAT pVal	eWAT FC	eWAT pVal
Slxl1	17534813	666135; 75140	-1,37	0,7177	-1,62	**0,0433**	**-3,32**	**0,0007**
Acly	17269521	104112	-1,1	0,2555	1,29	**0,0131**	**2,47**	**3,47E-07**
Ssty2	17546498	70009	-1,4	0,8732	-1,63	0,1748	**-3,29**	**0,007**
Gm20854	17547456	100040911	-1,29	0,9146	-1,55	0,1929	**-3,17**	**0,0088**
Acss2	17378359	60525; 105244340	-1,81	**0,0002**	1,09	0,2107	**2,06**	**5,42E-05**
Gm20852	17547288	100040786	-1,3	0,8875	-1,6	0,1697	**-3,29**	**0,0056**
Mrgpra9	17491315	668725	-1,2	0,3884	1,86	**0,0085**	**-2,27**	**0,0099**
Aplnr	17372725	23796	-1,64	**0,0348**	-1,95	**0,0479**	**-2,1**	**0,0048**
Elovl6	17402558	170439	1,06	0,6438	1,19	0,1029	**2,22**	**9,49E-06**
Serpina3b	17278261	271047	-1,02	0,7879	-1,11	0,3493	**-4,1**	**6,12E-05**
Hist1h2bm	17285683	319186	1,17	0,309	-1,39	0,0722	**-2,09**	**1,01E-05**
Adora1	17227094	11539	-1,46	**0,0062**	1,75	**0,0003**	**2,04**	**7,86E-07**
Gm20736	17546588	380994; 10086183[illegible]	-1,2	0,8244	-1,8	0,0914	**-4,51**	**0,0004**
Vmn1r58	17486039	81014	-1,1	0,8371	1,25	0,536	**-2,06**	**0,0004**
Olfr591	17494160	258139	-1,24	0,8006	-1,28	0,3233	**-2,1**	**0,0046**
Gm21440	17546415	100040223; 10086[illegible]	-1,12	0,7586	-1,52	0,1869	**-2,65**	**0,0087**

Supplementary Table S7. List of transcription factors predicted by CheA and TRRUST for the different modules of iWAT.

Excel file

Supplementary Table S8. Quantification by qPCR of the expression pattern of key transcription factors putatively regulating genes from modules I and II. n=4/group for iWAT and eWAT and n=3/group for iBAT.

		iBAT		iWAT		eWAT	
**Module**	**Gene Symbol**	**Fold change**	**p-value**	**Fold change**	**p-value**	**Fold change**	**p-value**
Module I	*Ppara*	-1,05	0,8700	6,94	0,0001	1,22	0,0812
	Pparg	-1,48	0,0823	1,13	0,6408	1,46	0,2064
	Pgc1a	3,12	0,0124	9,45	0,0012	4,30	0,0477
Module II	*Pu.1*	-1,30	0,4466	-2,26	0,0673	1,03	0,8121

Supplementary Table S9. List of miRNAs specifically upregulated and downregulated in the iWAT depot. Table indicating fold change and p-values in the iBAT, iWAT, and eWAT of all the miRNAs upregulated or downregulated specifically in the iWAT depot. Fold change values higher than 2 are coloured in green, while fold change values lower than -2 in blue. FC; Fold-change. pVal; p-value.

ID	Transcript ID	iBAT FC	iBAT pVal	iWAT FC	iWAT pVal	eWAT FC	eWAT pVal
20500648	mmu-miR-300-3p	1,36	0,8785	**-13,91**	**0,0109**	1,15	0,892
20504677	mmu-miR-497-5p	-1,11	0,237	**-9,46**	**0,0267**	1,04	0,9473
20500956	mmu-miR-329-3p	1,52	0,865	**-7,57**	0,0913	-1,68	0,4682
20525998	mmu-miR-7016-3p	-1,5	0,3542	**-6,58**	0,1227	1,43	0,7858
20505073	mmu-miR-676-3p	-1,35	0,2462	**-5,43**	**0,0261**	-1,18	0,9535
20506737	mmu-miR-466f	-1,57	0,151	**-4,76**	**0,0004**	1,29	0,4853
20500995	mmu-miR-342-5p	1,08	0,8679	**-4,39**	**0,0006**	-1,19	0,3809
20504680	mmu-miR-423-3p	1,07	0,421	**-4,32**	0,2244	1,2	0,7323
20500397	mmu-miR-199a-5p	-1,11	0,2157	**-4,17**	0,2496	1,53	0,4632
20500662	mmu-miR-106b-3p	1,51	0,7615	**-4,01**	0,1105	-1,28	0,6163
20500916	mmu-miR-34a-5p	1,22	0,2226	**-3,9**	0,0596	1,09	0,6705
20504745	mmu-miR-500-3p	-1,14	0,3429	**-3,76**	**0,0011**	-1,87	0,4893
20504624	mmu-miR-1843a-5p	-1,37	0,7084	**-3,71**	0,1165	1,17	0,579
20500297	mmu-miR-145a-5p	1,02	0,1919	**-3,51**	**0,0219**	1,13	0,7036
20525878	mmu-miR-6958-3p	-1,07	0,9397	**-3,44**	**0,0307**	1,47	0,7325
20500873	mmu-let-7b-3p	1,37	0,9502	**-3,36**	**0,0051**	1,21	0,5821
20500376	mmu-miR-187-3p	-1,68	0,6196	**-3,35**	0,1197	1,35	0,472
20501791	mmu-miR-376b-3p	-1,44	0,8823	**-3,33**	**0,0014**	1,07	0,9936
20500299	mmu-miR-146a-5p	-1,25	0,269	**-3,28**	**0,0003**	-1,02	0,9289
20520355	mmu-miR-5100	-1,53	0,1066	**-3,21**	0,0766	-1,15	0,9206
20500363	mmu-miR-181a-5p	-1,24	0,0998	**-3,08**	0,1142	1,02	0,7117
20500907	mmu-miR-27a-3p	-1,11	0,705	**-3,05**	0,0564	1,06	0,3799
20501145	mmu-miR-125b-1-3p	-1,03	0,8023	**-2,89**	**0,0162**	1,91	0,4123
20500977	mmu-miR-148b-3p	-1,2	0,2294	**-2,88**	0,115	-1,03	0,9759
20500653	mmu-miR-34c-5p	1,12	0,9597	**-2,87**	0,1279	1,41	0,4952
20500392	mmu-miR-195a-3p	-1,35	0,0875	**-2,84**	**0,0038**	-1,4	0,4804
20501203	mmu-miR-362-5p	1,01	0,9933	**-2,8**	**0,0059**	-1,37	0,3561
20506730	mmu-miR-669f-5p	-1,33	0,3564	**-2,71**	0,467	-1,34	0,4684
20504709	mmu-miR-669c-5p	1,56	0,4469	**-2,61**	0,0631	1,63	0,2091
20538080	mmu-mir-8112	-1,22	0,3456	**-2,58**	**6,36E-05**	1,15	0,4147
20501141	mmu-miR-181b-5p	-1,97	0,0556	**-2,56**	**0,0032**	-1,4	0,7017
20515427	mmu-miR-466m-5p	1,31	0,8497	**-2,42**	**0,0226**	-1,82	0,0892
20510757	mmu-miR-669m-5p	1,31	0,8497	**-2,42**	**0,0226**	-1,82	0,0892
20505714	mmu-miR-466f-5p	-1,36	0,485	**-2,42**	0,126	-1,82	0,2649
20500913	mmu-miR-93-3p	-1,97	0,529	**-2,4**	0,1252	1,3	0,9429
20503909	mmu-miR-532-5p	-1,27	0,5539	**-2,33**	**0,0122**	-1,35	0,9279
20506741	mmu-miR-466j	1,26	0,46	**-2,3**	0,1075	1,15	0,6885
20528534	mmu-miR-7658-5p	1,59	0,387	**-2,3**	0,2936	1,75	0,1908
20500398	mmu-miR-199a-3p	1,01	0,3952	**-2,28**	0,1128	1,51	0,5895
20501136	mmu-miR-199b-3p	1,01	0,3952	**-2,28**	0,1128	1,51	0,5895
20501094	mmu-miR-25-3p	-1,09	0,3926	**-2,25**	0,2716	-1,04	0,7829
20504627	mmu-miR-665-3p	1,01	0,9463	**-2,24**	**0,0005**	1,18	0,2601
20500642	mmu-miR-297a-5p	1,26	0,4213	**-2,23**	**0,0014**	1,38	0,325
20526098	mmu-miR-7066-3p	1,2	0,9822	**-2,22**	**0,0154**	1,07	0,8923
20505702	mmu-miR-421-3p	-1,56	0,4078	**-2,22**	**0,033**	-1,08	0,7296
20504226	mmu-miR-487b-3p	-1,62	**0,0305**	**-2,17**	0,0569	-1,28	0,8784
20504162	mmu-miR-486-3p	1,01	0,9692	**-2,14**	0,6873	1,74	0,2014
20501302	mmu-miR-335-5p	1,01	0,8683	**-2,13**	0,3746	1,02	0,7412

20501017	mmu-miR-350-3p	-1,95	0,4394	**-2,12**	0,4443	1,13	0,7697
20500305	mmu-miR-151-5p	-1,15	0,3025	**-2,12**	0,5367	-1,11	0,8263
20501787	mmu-miR-409-3p	1,22	0,2983	**-2,07**	**0,0056**	1,17	0,5811
20500911	mmu-miR-92a-3p	-1,02	0,2897	**-2,07**	0,3643	1,07	0,5725
20504200	mmu-miR-541-5p	-1,28	0,6372	**-2,06**	**0,0047**	-1,34	0,612
20504144	mmu-miR-483-5p	-1,48	0,0759	**2,02**	0,1293	1,51	0,067
20505777	mmu-miR-877-5p	1,91	0,2334	**2,03**	0,149	1,4	0,2412
20501097	mmu-miR-32-5p	-1,04	0,6048	**2,09**	**0,0065**	-1,13	0,5632
20515372	mmu-miR-3060-3p	1,27	0,9599	**2,12**	**0,0484**	1,64	0,7115
20525907	mmu-miR-6972-5p	1	0,7956	**2,23**	**0,0238**	1,19	0,0683
20525789	mmu-miR-6914-5p	-1,37	0,8106	**2,27**	0,1	-1,05	0,8584
20525979	mmu-miR-7007-5p	1,14	0,6492	**2,29**	0,2739	1,56	0,2875
20506725	mmu-miR-466i-3p	-1,03	0,7601	**2,4**	0,1087	-1,72	0,1097
20525757	mmu-miR-6898-5p	1,14	0,8258	**2,51**	0,105	1,31	0,7058
20500278	mmu-miR-133a-3p	-1,05	0,6201	**2,53**	0,1189	-1,06	0,7597
20504599	mmu-miR-1247-3p	1,22	0,7709	**2,62**	0,0722	-1,3	0,4145
20500240	mmu-miR-1a-3p	-1,08	0,8341	**3,03**	0,0981	1,3	0,6983
20529965	mmu-miR-8102	1,71	0,5921	**3,32**	**0,0268**	-1,19	0,6761
20516315	mmu-miR-3473a	-1	0,7708	**3,54**	**0,0061**	1,39	0,4551
20501308	mmu-miR-133b-3p	-1,25	0,5744	**4,65**	0,0626	-1,15	0,9561

Supplementary Table S10. Regulatory interactions between miRNAs and genes of the iWAT network. Table indicating the interactions occurring between miRNAs and genes specifically upregulated and downregulated in the iWAT depot. Fold change values higher than 2 are coloured in green, while fold change values lower than -2 in blue.

miRNAs		Genes	
**miRNA_ID**	**Fold Change**	**GeneSymbol**	**Fold Change**
mmu-miR-145a-5p	**-3,51**	Ms4a6c	**-2,6**
mmu-miR-145a-5p	**-3,51**	Cox15	**2,36**
mmu-miR-148b-3p	**-2,88**	Ucp3	**2,94**
mmu-miR-181b-5p	**-2,56**	Ppara	**3,82**
mmu-miR-1843a-5p	**-3,71**	Fstl1	**-2,95**
mmu-miR-1843a-5p	**-3,71**	Tomm40	**2,64**
mmu-miR-27a-3p	**-3,05**	Ctsc	**-2,14**
mmu-miR-27a-3p	**-3,05**	Pank1	**4,7**
mmu-miR-297a-5p	**-2,23**	Srl	**2,01**
mmu-miR-297a-5p	**-2,23**	Xrcc3	**2,12**
mmu-miR-329-3p	**-7,57**	Ppp1r3b	**2,78**
mmu-miR-342-5p	**-4,39**	Gnao1	**2,83**
mmu-miR-342-5p	**-4,39**	Pank1	**4,7**
mmu-miR-342-5p	**-4,39**	Vcan	**-3,05**
mmu-miR-409-3p	**-2,07**	Esrrg	**2,05**
mmu-miR-466f	**-4,76**	Acot2	**3,53**
mmu-miR-466i-3p	**2,4**	Ehhadh	**4,21**
mmu-miR-466i-3p	**2,4**	Msr1	**-2,24**
mmu-miR-466i-3p	**2,4**	Herc6	**-2,27**
mmu-miR-466j	**-2,3**	Acot2	**3,53**
mmu-miR-466j	**-2,3**	Cox15	**2,36**
mmu-miR-466m-5p	**-2,42**	Acot2	**3,53**
mmu-miR-466m-5p	**-2,42**	Cox15	**2,36**
mmu-miR-500-3p	**-3,76**	F13a1	**-2,16**
mmu-miR-500-3p	**-3,76**	Lair1	**-2,14**
mmu-miR-500-3p	**-3,76**	Prkaa2	**2,41**
mmu-miR-665-3p	**-2,24**	Slc2a4	**2,05**
mmu-miR-665-3p	**-2,24**	Cox10	**2,59**
mmu-miR-665-3p	**-2,24**	Gnao1	**2,83**
mmu-miR-665-3p	**-2,24**	Ppara	**3,82**
mmu-miR-665-3p	**-2,24**	Prkaa2	**2,41**
mmu-miR-669m-5p	**-2,42**	Acot2	**3,53**
mmu-miR-669m-5p	**-2,42**	Cox15	**2,36**
mmu-miR-6898-5p	**2,51**	B4galt6	**-2,24**
mmu-miR-6972-5p	**2,23**	Slc2a4	**2,05**
mmu-miR-6972-5p	**2,23**	Vcan	**-3,05**
mmu-miR-7007-5p	**2,29**	Adra1a	**2,31**
mmu-miR-7007-5p	**2,29**	Chrdl1	**-2,43**
mmu-miR-7007-5p	**2,29**	Fcgr1	**-3,21**
mmu-miR-7007-5p	**2,29**	Zadh2	**2,33**
mmu-miR-7658-5p	**-2,3**	Decr2	**2,03**
mmu-miR-7658-5p	**-2,3**	Ptges2	**2,22**
mmu-miR-877-5p	**2,03**	Chrdl1	**-2,43**
